# Stringent response ensures the timely adaptation of bacterial growth to nutrient downshift

**DOI:** 10.1038/s41467-023-36254-0

**Published:** 2023-01-28

**Authors:** Manlu Zhu, Xiongfeng Dai

**Affiliations:** Hubei Key Laboratory of Genetic Regulation and Integrative Biology, School of Life Sciences, Central China Normal University, Wuhan, Hubei Province China

**Keywords:** Bacterial physiology, Bacteriology, Bacterial systems biology, Bacterial transcription

## Abstract

Timely adaptation to nutrient downshift is crucial for bacteria to maintain fitness during feast and famine cycle in the natural niche. However, the molecular mechanism that ensures the timely adaption of bacterial growth to nutrient downshift remains poorly understood. Here, we quantitatively investigated the adaptation of *Escherichia coli* to various kinds of nutrient downshift. We found that *relA* deficient strain, which is devoid of stringent response, exhibits a significantly longer growth lag than wild type strain during adapting to both amino acid downshift and carbon downshift. Quantitative proteomics show that increased (p)ppGpp level promotes the growth adaption of bacteria to amino acid downshift via triggering the proteome resource re-allocation from ribosome synthesis to amino acid biosynthesis. Such type of proteome re-allocation is significantly delayed in the *relA*-deficient strain, which underlies its longer lag than wild type strain during amino acid downshift. During carbon downshift, a lack of stringent response in *relA* deficient strain leads to disruption of the transcription-translation coordination, thus compromising the transcription processivity and further the timely expression of related catabolic operons for utilizing secondary carbon sources. Our studies shed light on the fundamental strategy of bacteria to maintain fitness under nutrient-fluctuating environments.

## Abstract (web summary)

Bacteria undergo nutrient fluctuations during repeated feast and famine cycles and need to metabolically adapt to these changes. Using quantitative proteomics, Zhu & Dai show that the stringent response of (p)ppGpp is crucial for the timely adaption of bacterial growth to both amino acid and carbon downshift.

## Introduction

The nutrient composition in the natural niche of bacteria is often highly fluctuating, leading to the so-called “feast and famine cycle”^1–5^. Nutrient-limited conditions could significantly affect the physiological property of bacteria such as increasing drug tolerance^6,7^. For enteric bacteria *Escherichia coli* living in the mammalian intestine, the availability and type of nutrient sources including amino acids and carbon sources vary substantially at different locations of intestinal tract (e.g., intestinal mucus, luminal content) and different time points of the daily life of the hosts^1,8,9^. It has been estimated that the average generation time of *E. coli* in intestine ranges from 20–30 min (the same as in rich nutrient broth) to over 1 day^1^. Therefore, bacterial cells must timely adapt their growth to changing nutrient environments in order to maintain population fitness. During nutrient transition, bacteria must reshape the global gene expression pattern and metabolome including upregulating the expression of required proteins as well as down-regulating the expression of non-required proteins to meet the metabolic requirement^10–14^. For example, compared with cells growing in rich medium, cells growing in poor nutrient conditions synthesize more metabolic proteins at the expense of less ribosomes^15–21^. It has recently been shown that during growth transition from various glycolytic carbons to the fermentation product, acetate, *E. coli* must re-program its metabolic landscape from glycolysis to glyoxylate shunt-gluconeogenesis pathway^11^, imposing a physiological bottleneck for the adaptation to acetate downshift. In such case, the physiological status of bacteria has a profound impact on the lag during acetate downshift^11^. A very recent study has further found that the proteome constraint from the expression of non-required proteins affects the lag time of *E. coli* during glucose-acetate diauxic growth^14^. These studies raise the important issue regarding the molecular players that control the growth adaptation to nutrient downshift. During sudden nutrient downshift events, bacteria generally require a certain lag time before resuming growth in the secondary nutrient conditions^11,22^, then what’s the molecular player that determines the lag time of nutrient downshift? Do there exist some global molecular strategies that ensure the timely adaption of bacterial growth to nutrient downshift? Uncovering the regulatory mechanism of growth adaption is of high importance for understanding the fundamental design principle of the bacterial system.

It is known that nutrient stress triggers a drastic accumulation of the alarmone guanosine tetra- or penta-phosphate [(p)ppGpp] inside bacteria cells. This phenomenon, collectively referred to as “stringent response” is proposed to be highly conserved throughout the bacterial domain of life^23–26^. (p)ppGpp-mediated stringent response orchestrates the re-programming of bacterial transcriptome^27^ as well as an incredibly diverse set of other biochemical processes during nutrient stress^23,26^. A major effect of (p)ppGpp is regulating transcription initiation via directly binding to RNA polymerase (RNAP) with the assistance of DksA, altering the expressions of hundreds of genes—some positively (e.g., amino acid biosynthesis genes), some negatively (e.g., *rrn* and ribosomal protein genes)^28–30^. During nutrient starvation, (p)ppGpp-mediated stringent response shuts down many biochemical processes of central dogma such as replication, rRNA transcription, ribosome maturation and translation initiation/elongation, resulting in growth arrest and promoting cell to enter into a dormant, non-growing state for surviving^26,31^. Previous studies have shown that the *relA* relaxed mutant displays a longer lag during transition from the stationary phase into the new exponential phase^32^or during metabolic inhibition by serine + methionine + glycine (SMG)^33^, suggesting that (p)ppGpp participates in regulating bacterial growth transition. Nevertheless, it remains elusive regarding the mechanistic connection between (p)ppGpp and growth adaption of bacteria to nutrient downshift.

In this work, we systematically explore the relation between (p)ppGpp and the growth adaption of bacteria to nutrient downshift (transition from a high-quality nutrient condition to a low-quality nutrient condition). We demonstrate that stringent response is crucial for the timely adaption of bacteria to both amino acid downshift (transition from rich medium to amino acid-free minimal medium) and carbon downshift (transition from the preferred carbon source, glucose to a poor secondary carbon source). Under amino acid downshift, (p)ppGpp promotes bacterial growth adaption via triggers a global resource re-allocation toward amino acid biosynthesis. Instead, (p)ppGpp promotes the bacterial growth adaption to carbon downshift via coordinating transcription with translation, further ensuring the transcription processivity and thus the timely synthesis of related catabolic proteins required for utilization of the secondary carbons.

## Results

### (p)ppGpp regulates the growth adaption of bacterial to amino acid downshift

**Fig. 1:**
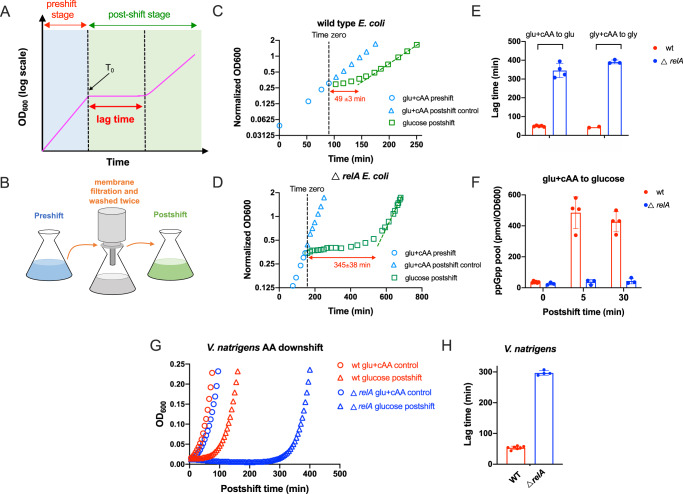
Growth curve and lag time of *E. coli* and *V. natriegens* during amino acid downshift (AA downshift). **A** Illustration of a typical bacterial growth curve of nutrient downshift. **B** Illustration of the culture-transfer protocol. The bacterial culture of preshift medium was collected by 0.22 µm filter membrane using filtration, washed twice by the postshift medium and then transferred to the postshift medium for culturing. The time cost of the whole culture-transfer process is generally within 3 min. **C** Growth curve and lag time of wild type *E. coli* cells during transition from glucose cAA medium (glu+cAA) to amino acid-free glucose minimal medium. **D** Growth curve and lag time of *E. coli relA*-deficient mutant during transition from glu+cAA medium to glucose minimal medium. **E** Comparison of lag time between wild type and *relA*-deficient *E. coli* cells during AA downshift. Both glucose (glu) medium and glycerol (gly) medium were used. Error bars are the standard deviations of several biological replicates (glu+cAA to glu: *n* = 5 and 4 for wild type and *relA*-deficient strain, respectively; gly to cAA to gly: *n* = 2 and 3 for wild type and *relA*-deficient strain, respectively). **F** The ppGpp pools of *E. coli* at 0 min, 5 min and 30 min during AA downshift. Error bars are the standard deviations of several biological replicates (*n* = 3 for *relA*-deficient mutant; for wild type strain, *n* = 6, 4 and 4 for 0 min, 5 min and 30 min, respectively. **G** Growth of wild type and *relA*-deficient *V. natriegens* strains after AA downshift (glucose as carbon source). OD_600_ during AA downshift in this case is automatically monitored by microplate reader. **H** Comparison of lag time between wild type and *relA*-deficient *V. natriegens* cells during AA downshift. Error bars are the standard deviations of several biological replicates (*n* = 7 and 4 for wild type and *relA*-deficient strain, respectively). Source data are provided as a Source Data file.

We first focused on the growth transition of *E. coli* K-12 NCM3722 strain from glucose casamino acid (cAA) medium to amino acid-free glucose minimal medium (amino acid downshift, briefly refers to as “AA downshift”). Bacteria cells first exponentially grew in the 1^st^ (preshift) medium (glucose cAA medium), and at time zero (T_0_), culture was quickly transferred to the 2^nd^ medium (glucose minimal medium) using filtration method^11^, entering into postshift stage (Fig. 1A, B). For wild type strain, cell growth was arrested immediately, and after a lag time of ~50 min (49 ± 3 min), *E. coli* resumed exponential growth in glucose minimal medium (Fig. 1C). To investigate the potential role of (p)ppGpp in regulating growth adaptation to AA downshift, we turned to *relA*-deficient strain, which is known to be devoid of stringent response during nutrient starvation^23^. Although *relA*-deficient strain has a comparable growth rate to wild type strain (supplementary Fig. 1), its lag time during AA downshift is much longer, reaching ~6 h (345 ± 38 min) (Fig. 1D, E). The same result was obtained when glycerol was used as the carbon source (Fig. 1E and Supplementary Fig. 2). Furthermore, we found that in contrast to the case of wild type strain (Fig. 1F, red), *relA*-deficient strain exhibits almost no change in cellular ppGpp pools during AA downshift (Fig. 1F, blue), confirming its deficiency of stringent response. We also performed similar studies on *Vibrio natriegens*, a marine bacterium known as the most rapidly replicating bacterium and has emerged as a next-generation genetic host of synthetic biology^34,35^. The deficiency of *relA* also substantially prolongs the lag time of *V. natriegens* during AA downshift (Fig. 1G, H). These results suggest an important role of (p)ppGpp in the adaptation of bacterial growth to AA downshift.

**Fig. 2:**
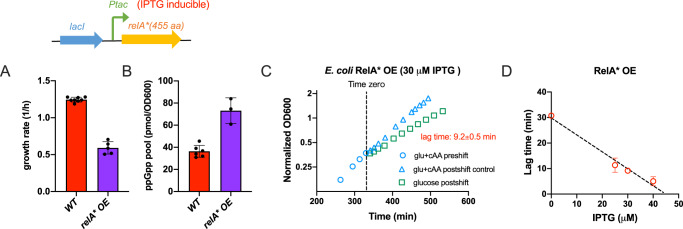
Growth adaption of *E. coli* to AA downshift during elevated (p)ppGpp level. *E. coli* was transformed with pLAS13 plasmids, in which a constitutively active relA* (encoding the N-terminal 1-455 aa of native *relA*) was driven by the IPTG-inducible *Ptac* promoter. **A** Effect of relA* overexpression (OE) on the growth rate of *E. coli* in glucose cAA medium. IPTG was added at a concentration of 30 µM. Error bars are the standard deviations of several biological replicates (*n* = 8 and 5 for wild type and relA* OE, respectively). **B** Effect of relA* overexpression on the cellular ppGpp pools of *E. coli* in glucose cAA medium. IPTG was added at a concentration of 30 µM. Error bars are the standard deviations of several biological replicates (*n* = 6 and 3 for wild type and relA* OE, respectively). **C** Growth of *E. coli* (pLAS13-relA*) strain during transition from glu+cAA medium to glucose minimal medium. IPTG was added at a concentration of 30 µM. **D** Lag time of *E. coli* (pLAS13-relA*) strain during AA downshift vs. IPTG concentration. Error bars are the standard deviations of several biological replicates (*n* = 3). Source data are provided as a Source Data file.

The long lag of the *relA-*deficient strain during AA downshift suggests (p)ppGpp has a positive role in bacterial growth adaptation to nutrient downshift. To further test this notion, we overexpressed the constitutively active RelA* in *E. coli* to induce the synthesis of intracellular (p)ppGpp^27,36,37^. As shown in Fig. 2, overexpression of RelA* (induced by 30 µM IPTG) reduces the growth rate but increases the cellular ppGpp pool (Fig. 2A, B). Strikingly, RelA* overexpression (RelA* OE) strain exhibits a much short lag (less than 10 min at 30 µM IPTG) during AA downshift (Fig. 2C), and the lag time is negatively correlated with the IPTG inducer level (Fig. 2D). Instead, the overexpression of a RelA’ protein defective in (p)ppGpp synthesis does not reduce the lag time^36^ (Supplementary Fig. 3), suggesting that it is (p)ppGpp overproduction that accelerates the adaptation rate. Collectively, these results strongly support a key role of (p)ppGpp in promoting the adaption of bacterial growth to AA downshift.

### (p)ppGpp regulates the proteome resource allocation of *E. coli*

**Fig. 3:**
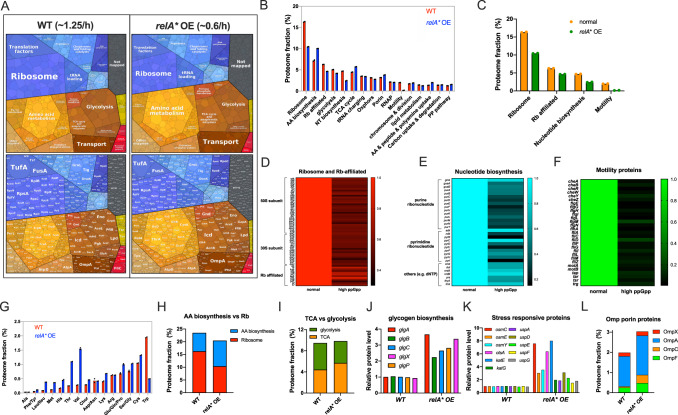
Global effect of (p)ppGpp on proteome resource allocation of *E. coli*. The proteome abundances of wild type strain (WT) and *E. coli* RelA* OE strain (pLAS13-relA*) during exponential growth in glucose cAA medium were investigated by 4D label-free mass spectrometry. Proteome fraction data are obtained from iBAQ intensity while relative abundances of individual proteins are based on LFQ intensity. The medium of *E. coli* RelA* OE strain was supplemented with 30 µM IPTG. This condition is thus referred to as “relA* OE” or “high ppGpp”. **A** Proteome resource allocation of *E. coli* analyzed by proteomaps. The proteome of *E. coli* was divided into various functional groups by proteomaps website based on KEGG classifications. **B** The absolute abundances of each proteome sector of *E. coli*. **C** Total abundances of ribosome proteins, ribosome (Rb)-affiliated proteins, nucleotide biosynthesis proteins and motility proteins of *E. coli*. **D**–**F** Relative abundances of individual proteins belong to “ribosome and Rb-affiliated”, “nucleotide biosynthesis” or “motility proteins”. **G** The proteome fractions of AA biosynthesis proteins of each AA subgroup. **H** The proteome fraction of ribosome proteins vs. that of AA biosynthesis proteins. **I** The proteome fraction of TCA vs. that of glycolysis. **J** Relative abundances of individual glycogen biosynthesis proteins. **K** Relative abundances of some typical stress responsive proteins. **L** The proteome fraction of the four “Omp” porin proteins. Data shown are the average of two biological replicates (*n* = 2). Source data are provided as a Source Data file.

When growing in poor nutrient (minimal medium), wild type *E. coli* cells generally harbor more metabolic proteins at the expense of fewer ribosomes than their counterparts growing in rich medium^15–21^. Following this logic, the slower growth of *E. coli* during (p)ppGpp overproduction here could mimic the effects of poor nutrient conditions, thus facilitating the adaption of *E. coli* to AA downshift. To test this scenario, we sought to explore the detailed impact of (p)ppGpp on the bacterial cellular resource allocation using quantitative proteomics as proteins carry out most of the cellular functions^38^. The proteome of exponentially growing *E. coli* was characterized by mass spectrometry including two conditions: (1) the standard condition (wild type strain growing in glu+cAA medium, growth rate:1.25/h), (2) the condition of (p)ppGpp induction (RelA* OE strain growing in glu+cAA with 30 µM IPTG, growth rate: 0.6/h). Our 4D-label-free proteomic approach captures ~2600 individual proteins of *E. coli* (Supplementary Data 1–3) and is of high reproducibility (Supplementary Fig. 4). With the results of LFQ intensity and iBAQ intensity given by Maxquant^39^, we could gain the information of both the relative abundances and absolute abundances of each protein (Supplementary Data 1–3). From a first glimpse with proteomaps (https://proteomaps.net)^40^ (Fig. 3A), it is clear to see a substantial decrease in the abundance of ribosome sector but a significant increase in the abundance of amino acid biosynthesis sector during (p)ppGpp overproduction (Fig. 3A), being consistent with the recent transcriptome study^27^.

Going beyond visualization, we next quantified the proteome fraction of various functional sectors (Fig. 3B and Supplementary data 3). Among them, the abundances of four categories including ribosome, Rb-affiliated proteins (such as EF-Tu, EF-Ts and EF-G), nucleotide biosynthesis proteins and motility proteins are most significantly affected by (p)ppGpp overproduction (Fig. 3C to 3F and Supplementary Data 3). In details, ribosome together with Rb-affiliated proteins, the most abundant proteome sectors, exhibit the largest decreasing trend during (p)ppGpp overproduction with regard to proteome fraction (drops from 22.6% to 15%) (Fig. 3C, D). The proteome fraction of nucleotide biosynthesis (mainly ribonucleotide biosynthesis) drops by a factor of two (from 4.7% to 2.4%), coinciding with a lower demand for rRNA (ribosome) synthesis (Fig. 3C,  E). Interestingly, (p)ppGpp is also known to inhibit nucleotide biosynthesis via directly targeting related key enzymes such as the inosine-guanosine kinase, Gsk^41^. Therefore, the negative effect of (p)ppGpp on nucleotide biosynthesis is achieved by reducing both the protein levels and enzymatic activities. Strikingly, (p)ppGpp overproduction causes a ~90% drop in the proteome fraction of motility proteins (from 2% to only 0.155%) (Fig. 3C, F), suggesting that the motility behavior of *E. coli* is inhibited together with the growth rate.

It is well established that (p)ppGpp could activate the expression of many amino acid promoters via either direct effect on RNA polymerase in combination with DksA or indirect effect resulting from releasing RNAP from rRNA promoters^29^. Being consistent with the picture, we do see that (p)ppGpp induction triggers an upregulation in most amino acid biosynthesis sub-groups (Fig. 3G, Supplementary Fig. S5, Supplementary Data 3). Quantitatively, the proteome fraction of the whole-AA biosynthesis sector increases from ~7% to 10% during (p)ppGpp induction (Fig. 3B and Supplementary Data 3). Hence, (p)ppGpp overproduction significantly increases the ratio of AA biosynthesis over ribosomes (Fig. 3H), confirming that (p)ppGpp overproduction indeed promotes the bacteria proteome into a status that is close to the poor nutrient condition as shown previously^15,18,19,38^. In addition, we also see an increase in the ratio of TCA over glycolysis (Fig. 3I), an indicator of saving energy and increasing the efficiency of carbon utilization by *E. coli*^38,42^. Besides all of this, (p)ppGpp also increases the expression of many other types of proteins such as glycogen biosynthesis proteins (for energy storage)^43^, stress responsive proteins^44,45^ and porin proteins^46^ (Fig. 3J to 3L), which are likely to be related to their physiological roles in bacterial stress survival. Overall, the high-resolution proteomic data here reveal a fine picture of the global effect of (p)ppGpp on bacterial cellular resource allocation.

### Dynamics of *E. coli* proteome during AA downshift

**Fig. 4:**
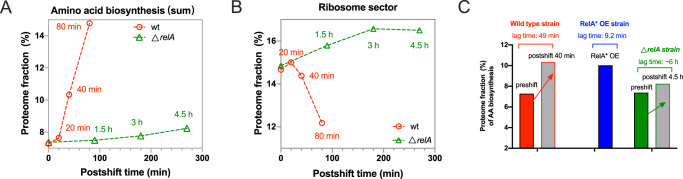
Dynamic change in the abundances of amino acid biosynthetic proteins during amino acid (AA) downshift. Wild type and *relA*-deficient *E. coli* strains undergo transition from glucose cAA medium to glucose minimal medium as shown in Fig. 1C, D. The proteomes of wild type cells at 0 min, 20 min, 40 min, and 80 min of postshift were analyzed while the proteomes of *relA*-deficient strain at 0 min, 1.5 h, 3 h, and 4.5 h of postshift were analyzed. **A** Dynamic change in the proteome abundance of the whole-AA biosynthesis sectors. **B** Dynamic change in the proteome abundance of the ribosome sector. **C** The proteome fraction of AA biosynthesis (${\phi }_{{AA}}$) at 40 min of postshift is ~10% for wild type strain. Coincidently, ${\phi }_{{AA}}$ of the RelA* OE strain during steady-state growth (blue bar, as done in Fig. 3) is also ~10%. For *relA*-deficient strain,$\,{\phi }_{{AA}}$ is 8% even at 4.5 h of postshift. Source data are provided as a Source Data file.

Quantitative proteomic studies above demonstrate that increased (p)ppGpp level triggers a proteome resource re-allocation of *E. coli* from ribosome synthesis to amino acid biosynthesis, coinciding with the metabolic demand of adaption to AA downshift and naturally explaining why (p)ppGpp induction accelerates the growth adaption of *E. coli* to AA downshift (Fig. 2). To further explore the scenario, we compared the time-course proteomes of wild type and *relA*-deficient strain during AA downshift (Supplementary Data 4–6). Wild type strain exhibits a rapid and strong upregulation in the amino acid biosynthesis sector and also a downregulation in ribosome sector during AA downshift (Red in Fig. 4A, B and Supplementary Fig. 6). In contrast, the response of amino acid biosynthesis pathways in the *relA*-deficient strain (green in Fig. 4A, B) is much slower than that of wild type strain, and moreover, the less-required ribosome sector (in minimal medium) even slightly increases in the *relA*-deficient strain. Therefore, the resource re-allocation from ribosome synthesis to amino acid biosynthesis, that is required for the metabolic adaption to AA downshift, is significantly delayed in *relA*-deficient strain due to lack of stringent response control.

Quantitatively, the proteome fraction of amino acid biosynthesis sector (${\phi }_{{AA}}$) in wild type strain increases from ~7.3% to ~10.3% at 40 min of postshift (Fig. 4A, red). During this period, the change in the proteome fraction of ribosome sector (${\phi }_{{Rb}}$) is marginal (Fig. 4B, red, from 14.7% to 14.4%), suggesting that the increase of ${\phi }_{{AA}}$ during this period is important for the adaption to AA downshift. For the *relA*-deficient strain, ${\phi }_{{AA}}$ only changes from ~7.4% to 8.2% in *relA*-deficient strain even after 4.5 h (Fig. 4A, green). Importantly, the ${\phi }_{{AA}}$ is ~10% in the case of (p)ppGpp induction via RelA* OE (Fig. 3B, C), which corresponds to a lag time of ~9 min (Figs. 2C and 4C). Coincidently, the ${\phi }_{{AA}}$ for wild type strain at 40 min of postshift during AA downshift is also ~10%, and exponential growth of wild type strain is also completely resumed after a further 9 min (lag time: 49 ± 3 min, Fig. 4C). Accordingly, for *relA*-deficient strain during 4.5 h of postshift, a ${\phi }_{{AA}}$ of 8.2% is not enough for resuming exponential growth (lag time~6 h, Fig. 1D, E). qRT-PCR of several amino acid biosynthesis genes suggests that the delayed synthesis of amino acid biosynthesis proteins in *relA*-deficient strain occurs at the level of transcription initiation (Supplementary Fig. 7), being consistent with the effect of DksA-(p)ppGpp in activating the transcription initiation of amino acid biosynthesis promoter^27–30^. Taken together, we conclude that (p)ppGpp-mediated stringent response ensures the timely growth adaption of bacteria to AA downshift via triggering bacterial resource re-allocation toward amino acid biosynthesis.

### (p)ppGpp regulates the growth adaption of bacterial to carbon downshift

**Fig. 5:**
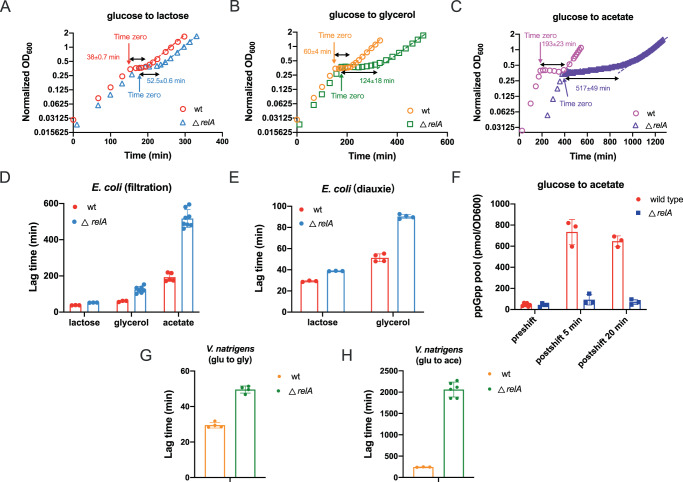
Growth curve and lag time of *E. coli* and *V. natriegens* during carbon downshift. **A** Growth and lag of *E. coli* wild type and *relA*-deficient strains during transition from glucose medium to lactose medium. **B** Growth and lag of *E. coli* wild type and *relA*-deficient strains during transition from glucose medium to glycerol medium. **C** Growth and lag of *E. coli* wild type and *relA*-deficient strains during transition from glucose medium to acetate medium. **D** Lag time of *E. coli* during carbon downshift using filtration method. Error bars are the standard deviations of several biological replicates (wild type strain: *n* = 3, 3, and 5 for lactose, glycerol, and acetate, respectively; *relA*-deficient strains: *n* = 3, 7, and 8 for lactose, glycerol, and acetate, respectively). **E** Lag time of *E. coli* during carbon diauxie, in which the secondary carbon source (lactose or glycerol) is supplemented together with glucose to the culture. Error bars are the standard deviations of several biological replicates (*n* = 3 for lactose; *n* = 4 for glycerol). **F** The ppGpp pools of *E. coli* at 0 min, 5 min, and 20 min after transition from glucose medium to acetate medium. Error bars are the standard deviations of several biological replicates (for 0 min condition, *n* = 6 and 4 for wild type and *relA*-deficient strain, respectively; n = 3 for the rest conditions). **G** Lag time of *V. natriegens* during glucose to glycerol transition. Error bars are the standard deviations of several biological replicates (*n* = 4). **H** Lag time of *V. natriegens* during glucose to acetate transition. Error bars are the standard deviations of several biological replicates (*n* = 3 and 6 for wild type and *relA*-deficient strain, respectively). Source data are provided as a Source Data file.

We next wondered whether (p)ppGpp also played a role in growth adaption to carbon downshift and thus performed a series of carbon downshift experiments. Glucose is the preferred carbon source of *E. coli*, and we had *E. coli* cultures shifted from glucose to three secondary carbon sources including lactose, glycerol and acetate, respectively (using filtration method) (Fig. 5A–C). In all the three types of carbon shift, *relA*-deficient strain exhibit significantly longer growth lags than wild type strain (Fig. 5A–D). The longer lags of *relA*-deficient strain than wild type cells are also observed during glucose-lactose and glucose-glycerol diauxie (the biphasic growth of microbes when growing on two carbon sources due to sequential utilization of carbon sources, Fig. 5E, Supplementary Fig. 8A, B), being consistent with previous reports^47,48^. In addition, stringent response is also largely abolished in *relA*-deficient strain during carbon downshift (Fig. 5F), suggesting that *relA* is also activated in stringent response during carbon downshift. This observation is consistent with a recent study showing that *relA* is also indirectly activated during fatty acid starvation resulting from depletion of pyruvate, an important precursor of lysine^49^. The longer lag of *relA*-deficient strain than wild type cells is also applicable to *V. natriegens* during both glycerol downshift (Fig. 5G and Supplementary Fig. 8C) and acetate downshift (Fig. 5H and Supplementary Fig. 8D). Therefore, (p)ppGpp also plays an important role in regulating the adaptation of bacterial growth to carbon downshift.

### Loss of transcription processivity of catabolic operons in *relA*-deficient strain during carbon downshift

**Fig. 6:**
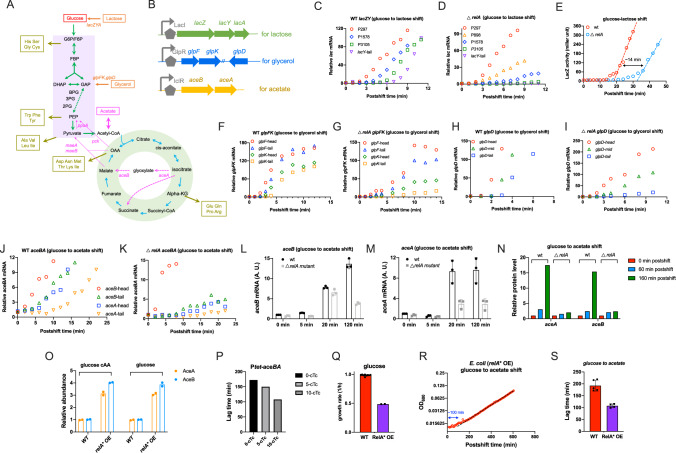
The induction kinetics of catabolic operons in *E. coli* during carbon downshift. **A** The central catabolic pathways of *E. coli* for related carbon sources. **B** Three catabolic operons. **C**, **D** The induction kinetics of *lac* mRNA in *E. coli* wild type strain and *relA*-deficient strain during glucose to lactose transition. **E** The induction kinetics of LacZ protein in *E. coli* wild type strain and *relA*-deficient strain during glucose to lactose transition. **F**, **G** The induction kinetics of *glpFK* mRNA in *E. coli* wild type strain and *relA*-deficient strain during glucose to glycerol transition. **H**, **I** The induction kinetics of *glpD* mRNA in *E. coli* wild type strain and *relA*-deficient strain during glucose to glycerol transition. **J**, **K** The induction kinetics of *aceBA* mRNA in *E. coli* wild type strain and *relA*-deficient strain during glucose to acetate transition. **L**, **M** The relative abundances (LFQ intensity) of *aceB* and *aceA* mRNA (detected by their 3’-tail region primers) of *E. coli* at different time points after shifting from glucose to acetate medium. Error bars are the standard deviations of three biological replicates (*n* = 3). **N** The relative abundances of AceA and AceB proteins of *E. coli* at different time points after shifting from glucose to acetate medium. **O** Effect of (p)ppGpp induction on the levels of AceA and AceB proteins. Overexpression of RelA* (Ptac-relA* induced by 30 µM IPTG) was used to increase the cellular (p)ppGpp pools of *E. coli*. **P** The effect of AceBA overexpression on the lag time of *E. coli* during glucose to acetate transition. Data originated from Balakrishnan et al.^14^; **Q** Growth rate of wild type strain and RelA* overexpression strain (30 µM IPTG) in glucose minimal medium. *n* = 9 and 2 for wild type and *relA*-deficient strain, respectively. **R** The growth and lag of RelA* overexpression strain (measured by microplate reader). **S** The effect of RelA* overexpression on the lag time of *E. coli*. Error bars are the standard deviations of at least four biological replicates (*n* = 5 and 4 for wild type and relA* OE, respectively). Source data are provided as a Source Data file.

During carbon downshift, bacteria must induce the expression of related catabolic operons for utilization of the 2^nd^ carbon in order to resume exponential growth. Like glucose, lactose and glycerol both belongs to glycolytic carbon sources, entering into central catabolic pathways via glycolysis and thus sharing the same metabolic route with glucose in vivo (Fig. 6A)^50^. Therefore, the key bottlenecks of adapting to lactose and glycerol downshift are the induction of *lac* operon and *glpFK, glpD* operons for utilization of lactose and glycerol, respectively (Fig. 6B)^22^. Unlike lactose and glycerol, utilization of acetate (a gluconeogenic carbon source) requires *E. coli* to activate the glyoxylate shunt (mediated by *aceBA* operon, Fig. 6B)^51^ and further reverse the metabolic route from glycolysis to gluconeogenesis to guarantee the ongoing synthesis of all necessary amino acid precursors (Fig. 6A)^11,14^. In accordance to this scenario, it has been recently shown that the absolute expression levels of AceA, AceB proteins strongly limit the growth transition of glucose-acetate diauxie^14^.

Following the above logic, we postulated that (p)ppGpp could exert its effect on growth adaption of *E. coli* to carbon downshift via affecting the induction of related catabolic operons. We thus quantified the mRNA induction kinetics of *lac* operon, *glpFK & glpD* operons and *aceBA* operon of *E. coli* during carbon downshift. For each catabolic operon, we designed three to five qPCR primers to detect different sub-regions of mRNA of related catabolic operons from 5’ head to 3’ end (Supplementary Information)^52,53^. The induction kinetics of 5’ head mRNA sub-region reflects the transcription initiation rate while comparing the induction kinetics of different mRNA sub-regions from 5’ head to 3’-tail allows us to obtain the status of transcription processivity^54^. A loss of transcription processivity, meaning that a portion of RNA polymerases fails to reach the 3’ end of mRNA to generate an intact mRNA product, suggests the occurrence of premature transcription termination (PTT)^52^. In such case, we should observe a significant drop of the mRNA accumulation rate from 5’ to 3’ direction.

We first looked at the induction of *lac* operon of *E. coli* during lactose downshift. The synthesis of *lac* mRNA was strongly induced (~100-fold) during lactose downshift (Fig. 6C). Moreover, the induction kinetics of each mRNA sub-regions from 5’ head of *lacZ* to 3’ tail of *lacY* were comparable with each other, suggesting a full transcription processivity. There is also a ~100-fold induction of 5’ head region of *lac* mRNA in *relA*-deficient strain (red, P297 primer, Fig. 6D), suggesting that transcription initiation is equally induced in wild type strain and *relA*-deficient strain. However, the accumulation rate of *lac* mRNA in *relA*-deficient strain displays a over 95% drop from 5’ head to 3’ tail of *lacZY* mRNA regions (Fig. 6D), a feature of the “polarity phenomenon”^55^, suggesting that transcription processivity of *lac* operon is severely compromised in *relA*-deficient strain during lactose downshift. Accordingly, the induction of LacZ protein exhibits a ~14 min delay in *relA*-deficient strain relative to wild type strain (Fig. 6E). We note that the ~14 min delay just coincides with the difference of lag time between wild type strain (~38 min) and *relA*-deficient strain (~52 min) (Fig. 5A). Hence, the longer lag of *relA*-deficient strain is attributed to the delayed expression of *lac* operon resulting from a loss of transcription processivity. Similarly, the polarity phenomenon also occurs strongly for both *glpFK* and *glpD* operons in *relA*-deficient strain (Figs. 6F, 6H for wild type; Fig. 6G, 6I for *relA*-deficient strain), being consistent with the finding of *lac* operon. Such a strong compromise in transcription processivity would certainly limit the timely synthesis of glycerol catabolic proteins, resulting in a longer lag time for *relA*-deficient strain (124 min) than wild type strain (60 min) during glycerol downshift (Fig. 5B).

We finally turned to acetate downshift. The levels of AceA and AceB proteins of wild type strain increase over 15-fold at 160 min after growth transition from glucose to acetate while the levels of gluconeogenesis proteins (including PckA, PpsA, MaeA, MaeB) change weakly at this stage (Supplementary Fig. 9 and Supplementary data 8). We then examined the induction kinetics of *aceBA* mRNA in wild type and *relA*-deficient strains during acetate downshift. Like the behaviors of *lac* operon and *glpFK/glpD* operons shown above, the transcription processivity of *aceBA* operon was also significantly compromised in the *relA*-deficient strain (Fig. 6J, K). Being consistent with this result, the expression levels of *aceBA* operon during the growth lag is much lower in *relA*-deficient strain than wild type strain at both mRNA (Fig. 6L, M) and proteins levels (Fig. 6N and Supplementary Data 8), further explaining the much longer lag of *relA*-deficient strain (~520 min) than wild type strain (~190 min) during acetate downshift (Fig. 5C). In addition, proteomic study shows that (p)ppGpp induction by RelA* overexpression increases the levels of AceA and AceB by three- to four-fold in both glucose cAA medium and glucose medium (Fig. 6O). It has been recently demonstrated that pre-induction of *aceBA* expression significantly shortens the growth lag of *E. coli* during glucose to acetate transition (Fig. 6P)^14^. Here we found that (p)ppGpp induction by RelA* overexpression, while reducing the exponential growth rate by 50% (Fig. 6Q), indeed shortens the lag time by nearly 50% (Fig. 6R, S). Taken together, the result of this section demonstrates that (p)ppGpp ensures the timely adaption of bacterial growth to carbon downshift via ensuring transcription processivity and the timely response of certain catabolic operons.

### (p)ppGpp assists transcription-translation coordination during nutrient downshift

What could be the mechanistic origin underlying the compromised transcription processivity of these catabolic operons in *relA*-deficient strain? We recall that the loss of transcription processivity (premature transcription termination, PTT) also occurs strongly in bacteria cells treated with translation-inhibitory drugs like fusidic acid and chloramphenicol, which disrupts the transcription-translation coordination^52^. It is well established that the elongation speed of RNAP and its trailing ribosome inside *E. coli* growing at normal growth conditions are maintained at the same value (30-50 nt/s and 10-17 aa/s, respectively)^52,54,56^, ensuring the tight coordination of transcription and translation. A disruption of transcription-translation coordination triggers Rho-dependent PTT, further compromising transcription processivity and the integrity of gene expression^52,55^.

**Fig. 7:**
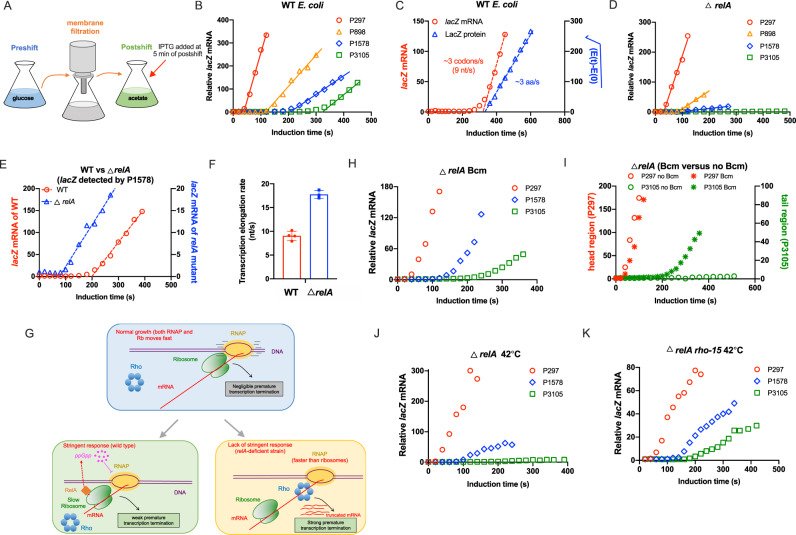
(p)ppGpp assists transcription-translation coordination in *E. coli* during carbon downshift. **A** Schematic illustration of the experimental design. **B** Induction kinetics of *lacZ* mRNA of wild type *E. coli* during glucose to acetate transition. **C** Induction kinetics of *lacZ* mRNA and LacZ protein of wild type *E. coli* during glucose to acetate transition. The transcription and translation elongation rates, deduced from the induction kinetics, are also shown. **D** The induction kinetics of *lacZ* mRNA of *E. coli relA*-deficient strain during glucose to acetate transition. **E** Comparison of the transcription kinetics of *lacZ* mRNA (detected by P1578 primers) between wild type strain and *relA*-deficient strain. **F** Transcription elongation rates of wild type strain and *relA*-deficient strain. Error bars are the standard deviations of several biological replicates (*n* = 4 and 3 for wild type and *relA*-deficient strain, respectively). **G** Schematics of the transcription-translation status of *E. coli*. During normal exponential growth, transcription and translation are tightly coordinated to maintain a full transcription processivity. Rho-mediated premature transcription termination (PTT) is negligible (panel **G**, upper). During carbon downshift, slow translation of ribosomes triggers the RelA-dependent stringent response of (p)ppGpp synthesis, further ensuring the maintenance of transcription-translation coordination and the full transcription processivity via (p)ppGpp’s effect on inhibiting transcription elongation (panel **G**, down, left). In the case of *relA*-deficient strain, transcription elongation (due to the lack of (p)ppGpp inhibition) is faster than that of translation elongation, leading to the disruption of transcription-translation coordination and furthermore the loss of transcription processivity by PTT. **H** The induction kinetics of *lacZ* mRNA of *E. coli relA*-deficient strain (treated with 25 μg/mL bicyclomycin, Bcm) during glucose to acetate transition. **I** Comparison of the induction kinetics of *relA*-deficient strain between no Bcm treatment case and Bcm-treated case. **J** The induction kinetics of *lacZ* mRNA of *E. coli relA*-deficient strain during glucose to acetate transition under 42 °C. **K** The induction kinetics of *lacZ* mRNA of *E. coli rho−15, relA*-deficient strain during glucose to acetate transition under 42 °C. Source data are provided as a Source Data file.

In light of the above point, we wondered whether the loss of processivity of these catabolic operons in *relA*-deficient strain was the result of a disruption of transcription-translation coordination. We then employed the recently established *lacZ* induction kinetics assay (for both mRNA and protein) to examine the status of transcription-translation coordination of *E. coli* during acetate downshift^52,53,57^. *E. coli* culture was first transferred from glucose medium to acetate medium and supplied with 5 mM IPTG to induce the *lacZ* expression at 5 min of postshift (Fig. 7A). As shown in Fig. 7B, the induction kinetics of each mRNA sub-region (detected by different primers) from 5’ head to 3’ tail of *lacZ* mRNA allows us to obtain the transcription elongation rate of *lacZ* mRNA. Meanwhile, the translation elongation rate of LacZ protein could be obtained with Schleif square-root plot analysis of the LacZ protein induction curve^58,59^. As shown in Fig. 7C, the synthesis of full-length *lacZ* mRNA and LacZ proteins take almost the same time (~320 s) for wild type strain during acetate downshift, suggesting that transcription-translation coordination is still maintained. Quantitatively, the absolute speeds of transcription and translation elongation are only ~9 nt/s and ~3 aa/s, respectively, which is only ~20% of the value of optimal condition (50 nt/s and 16–17 aa/s, respectively)^52^.

Unlike wild type strain, the induction kinetics of *lacZ* mRNA of *relA*-deficient strain again exhibits a substantial loss of transcription processivity without even generating any full-length *lacZ* mRNA (Fig. 7D, 3’ tail detected by P3105 primer), which is a hallmark of disruption of transcription-translation coordination^52,60^. Comparing the induction kinetics of middle sub-regions of *lacZ* mRNA (detected by P1578 primer) clearly show that the transcription elongation time required for *relA*-deficient strain is only half of that for wild type strain (Fig. 7E) so that the transcription elongation speed of *relA*-deficient strain (17.8 nt/s) is twice the value of wild type strain (9 nt/s) (Fig. 7F), being consistent with previous studies showing that (p)ppGpp could inhibit the transcription elongation rate under nutrient starvation conditions^60,61^. Since translation elongation rate is not affected in *relA*-deficient strain compared with wild type cells^58^, a strong disruption of transcription-translation coordination does occur in the *relA*-deficient strain.

Collectively, these results, together with the result of Fig. 6 supports the following picture as illustrated in Fig. 7G. During normal growth conditions, transcription and translation elongation are both fast and tightly coordinated with each other, ensuring a full transcription processivity and negligible level of PTT. During initial stage of carbon downshift, translation elongation rate drops substantially, triggering the RelA-dependent stringent response of (p)ppGpp synthesis^58,62,63^. The high (p)ppGpp pool then inhibits the elongation rate of RNA polymerase, ensuing the maintenance of transcription-translation coordination and further the transcription processivity of related catabolic operons. For *relA*-deficient strain in which stringent response is abolished, transcription elongation is much faster than translation elongation, leading to the disruption of transcription-translation coordination and further the Rho-mediated PTT. As a consequence, the transcription processivity of related catabolic operons is compromised, further limiting the timely synthesis of related carbon catabolic and uptake proteins. Nevertheless, with the basal expression of these certain catabolic operons, the 2nd carbon could still gradually enter into cells so that the non-coordinated status of transcription-translation will be ultimately alleviated in *relA*-deficient strain for resuming the utilization of 2nd carbon, after which the *relA*-deficient strain could resume growth. In such cases, lag time of *relA*-deficient strain is significantly longer than that of wild type strain during growth adaptation to carbon downshift.

Two further experiments were performed to test whether Rho is responsible for the loss of transcription processivity here. We first treated the *relA*-deficient strain with a sublethal dose of bicyclomycin (Bcm), a specific inhibitor of the Rho factor and performed the *lacZ* induction kinetics during acetate downshift. Compared with the no-drug treatment case (Fig. 7D), the induction of 3’ tail region of *lacZ* is indeed significantly recovered (~50-fold induction) by Bcm treatment (Fig. 7H, I). In another experiment, we generated the *rho*-15 polarity suppressor mutation in the *relA*-deficient background^64,65^. Rho-15 is a heat labile mutant version of Rho protein and 42 °C could significantly disable its function^64,65^. For *relA*-deficient strain, the polarity effect under 42 °C (Fig. 7J) is as strong as that under 37 °C (Fig. 7D). In contrast, the polarity effect is significantly alleviated in *rho*-15 *relA*-deficient strain, for which the induction kinetics of the 3’ tail region of *lacZ* becomes much more comparable to the 5’ head region (Fig. 7K). Therefore, these two results strongly support the occurrence of the loss of transcription processivity during carbon downshift is due to Rho-mediated PTT.

## Discussion

**Fig. 8:**
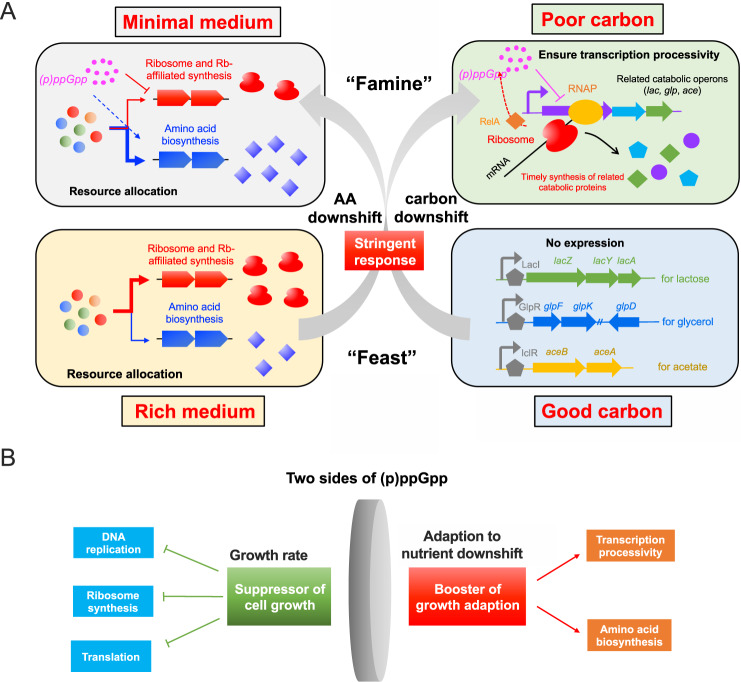
Schematic illustration of the role of (p)ppGpp in boosting the adaption of bacterial growth to nutrient downshift. **A** From “feast” to “famine”, stringent response plays an important role in ensuring the timely adaption of bacterial growth to nutrient downshift. During amino acid downshift (rich medium to minimal medium), (p)ppGpp promotes the growth adaption of bacterial via activating amino acid biosynthesis. In the case of carbon downshift, the RelA-dependent (p)ppGpp stringent response maintains the transcription-translation coordination and further ensures the transcription processivity so that related catabolic proteins of specific carbons (e.g., lactose, glycerol and acetate) could be timely synthesized to allow bacteria to resume growth in the 2nd carbon source. **B** The physiological role of (p)ppGpp is a coin of two sides. On one hand, (p)ppGpp acts as a global suppressor of cell growth via shutting down various biochemical processes such as DNA replication, ribosome synthesis, translation (initiation and elongation) during stringent response. On the other hand, as shown in our studies, the (p)ppGpp’s role in activating amino acid biosynthesis and maintaining transcription processivity (via coordinating transcription-translation) is crucial for the timely adaption of bacterial growth to nutrient downshift.

The capability of rapid adaptation to changing nutrient conditions is crucial for bacteria to maintain fitness and thrive in their natural niches. Uncovering the underlying regulatory mechanism of growth adaption is of great importance for understanding the design principle of bacterial system. In this work, we found that (p)ppGpp is a global regulator of the timely adaption of bacterial growth to nutrient downshift including both AA downshift and carbon downshift. As illustrated in Fig. 8, during AA downshift, (p)ppGpp-mediated stringent response re-allocates bacterial proteome resources from ribosome synthesis to amino acid biosynthesis, promoting the growth adaption of bacteria to minimal medium. Accordingly, *relA* deficiency (lack of stringent response) or artificial (p)ppGpp induction by RelA* overexpression decelerates or accelerates the growth adaptation of *E. coli* to AA downshift. Importantly, we found that (p)ppGpp also plays a key role in ensuring the growth adaption to carbon downshift from glucose to various carbon sources including lactose, glycerol and acetate. We show that (p)ppGpp exerts a distinctive and crucial effect here by coordinating transcription with translation during carbon downshift, further ensuring the transcription processivity and thus the timely induction of catabolic operons. The *relA*-deficient strain fails to tightly coordinate transcription with translation, displaying a strong polarity behavior in the induction of catabolic operons and requiring a longer time to adapt to the 2^nd^ carbon.

The synthesis of key metabolic proteins is delayed in *relA*-deficient strain during both AA downshift and carbon downshift; however, the underlying mechanistic origin likely differs from each other. (p)ppGpp is able to actively activate the amino acid biosynthesis (Figs. 3G & 4A, S5 to S7) at the level of transcription initiation, either by direct activating RNAP in combination with DksA or indirect effect resulting from increasing the availability of RNAP by releasing them from rRNA promoters^27,29^. Instead, during carbon downshift, (p)ppGpp functions on affecting the transcription processivity of catabolic operons by regulating the status of transcription-translation coordination, which occurs at the level of transcription elongation. Our study thus reveals a distinct form in which (p)ppGpp modulate the gene expression process beyond its effect on regulating transcription initiation, further exerting an important effect on bacterial fitness. In principle, the transcription processivity of amino acid biosynthesis operons could also be affected in the *relA* mutant during AA downshift. However, the slow induction kinetics of amino acid biosynthesis in the *relA* mutant during AA downshift makes it very difficult to quantify this effect (supplementary Fig. 7). Alternatively, transcription processivity of amino acid biosynthesis might not be affected as it is suggested previously that the effect of (p)ppGpp on transcription elongation depends on promoter and gene sequence^66^.

Mechanistically, the inhibitory effect of (p)ppGpp on transcription elongation could be either direct or indirect. In the scenario of direct mechanism, (p)ppGpp could directly bind to the elongating RNAP and increase its pausing frequency. Alternatively, (p)ppGpp could indirectly slow down RNAP via secondary effects such as inhibiting NTP biosynthesis^41^. Nevertheless, the negative effect of (p)ppGpp on transcription elongation rate could occur in few minutes during sudden nutrient downshift (Fig. 7)^60,61^, suggesting that a direct-binding mechanism is more likely to be the case. During transcription initiation of *E. coli*, (p)ppGpp modulate bacterial gene expression via binding to both the site 1 and site 2 of RNAP (binds to site 2 synergistically with DksA)^29^. In vitro study has shown that (p)ppGpp itself could directly cause RNAP pausing in the absence of DksA^67^, suggesting that the single binding of (p)ppGpp to site 1 contributes its effect on transcription elongation. In addition, DksA could further enhance such effect in vivo as there are evidences showing that DksA could also affect transcription elongation^68^.

Besides the key role of the maintenance of transcription processivity of catabolic operons, the timely downregulation of some less-required proteins (such as ribosomes) could also contribute the adaption to carbon downshift especially for the poor carbon, acetate, as it has been recently shown that proteome constraint from the expression of useless proteins also affects the lag time of diauxic transition^14^. In support of this scenario, we find that the downregulation of ribosome in *relA* mutant is also delayed compared with wild type strain (supplementary Fig. 10).

Growth is the fundamental property of bacterial cells. Growth is not just a narrow-sense term meaning “growth rate” but also has some other implications such as growth adaption to changing nutrients^11^ as growth kinetics often dynamically changes during the “famine to feast” cycle of bacteria in their natural niches. Our study demonstrates that the physiological role of (p)ppGpp in regulating cell growth is a coin of two sides (Fig. 8B). On one hand, it acts as a global suppressor of growth by shutting down various biochemical processes of central dogma during stringent response^23,31,69^. On the other hand, it acts as an engine of bacterial growth adaptation to nutrient downshift via activating amino acid biosynthesis during AA downshift and ensuring transcription processivity of catabolic operons during carbon downshift. In this sense, the stringent response not only promotes cells to enter into a “maintenance & survival mode” but also enables bacteria to be prepared for utilizing the lower-quality nutrients available in the environment.

Recent studies have suggested a trade-off relation between growth rate and other important traits in microbes such as growth rate vs. lag time^11,70–72^, growth rate vs. motility^42,73^. Evolution should in principle select bacterial species that could optimally balance these pairs of conflicting objectives according to their natural living conditions. Elucidating the mechanistic origin underlying these trade-off pairs of traits is crucial in understanding the diversity of bacterial phenotypes across conditions and species. Our study shows that (p)ppGpp signaling could act as a mechanistic origin underlying the trade-off between growth rate and growth adaption to nutrient downshift. Although (p)ppGpp induction slows down cell growth via inhibiting ribosome synthesis, it allows bacteria to more quickly adapt to AA downshift via activating amino acid biosynthesis. It is conceivable that for some types of bacterial species that lives in nutrient-limited and highly fluctuating environments, rapid adaption instead of rapid growth rate is selected, in which cases (p)ppGpp signaling could be modulated to obtain such trait.

## Methods

### Strains

Strains used in this study were mainly derivatives of *E. coli* K-12 NCM3722 strain or *V. natrigens* ATCC14048 strain^34^. *E. coli* strains used include wild type K-12 NCM3722 strain, *relA*-deficient strain, relA* OE strain (pLAS13-*Ptac*-relA*), the defective *relA’* OE strain (pLAS14-*Ptac*-*relA*’) and the *rho-15 relA::kan* mutant strain^37^. The *relA*-deficient strain was constructed by transforming the *relA::kan* locus of a MG1655 *relA*-deficient strain (a gift from Richard Gourse) to NCM3722 strain via P1 transduction. To construct a *rho*-15 mutant, the 3’ end sequence of *rho* ORF “tttcttcgaaatgatgaaacgctcataa” in *relA::kan* strain was replaced by “ggtaatgactccaacttattaa” so that the C-terminal residues of Rho proteins (DFFEMMKRS”) changed to “EVMTPTY”^65^. The replacement was based on Lambda-Red homologous recombination system with the chloramphenicol resistance gene as the selection marker^74^.

To construct a *V. natriegens relA*-deficient strain, the flanking region (-800 bp) upstream and downstream of *relA* was fused by overlapping PCR (Gold Green PCR mix, Tsingke Biotech) and inserted into the suicide plasmid pDM4^75^ using DH5 Lamda pir strain (kindly provided by Debra Milton and Qiuchun Li). The recombination plasmid was then first transformed into the ST18 pir donor strain^76^ and further transformed to *V. natriegens* for screen of single-crossover recombinant strain (LB3 medium plus 15 μg/mL Cm). The single-crossover strain was first incubated in antibiotic-free LB3 medium for 7–8 h at 37 °C and further transferred to LB3 + 10% sucrose medium for overnight incubation. Double crossover *relA* knockout strain was finally screened from LB3 + 10% sucrose plate and confirmed by PCR verification.

### Medium

*E. coli* was cultured in LB medium or routine M9 minimal medium (cold spring harbor protocol, NH_4_Cl as sole nitrogen source). Nutrient and carbon sources include 0.2% glucose plus 0.2% casamino acids (cAA), 0.2% glucose, 0.2% glycerol, 0.2% lactose and 60 mM acetate. *V. natriegens* was cultured in either LB3 medium (LB nutrient broth with a final 3% w/v sodium chloride) or M9 minimal medium (NH_4_Cl as sole nitrogen source) supplemented with different nutrient or carbon sources including 0.4% glucose and 0.4% casamino acids (cAA), 0.4% glucose, 0.4% glycerol and 60 mM acetate, respectively. The M9 minimal medium for *V. natriegens* contained an additional 2% w/v NaCl, as the same described in Lee et al.^34^.

### Cell growth

Cell growth procedures were performed in an air bath shaker (200 rpm) under 37 °C. A routine procedure for culturing exponential cells includes three steps: seed culture, pre-culture and final experimental culture. For seed culture, cells from a fresh colony were inoculated into LB broth for *E. coli* (LB3 for *V. natriegens*) and grew at 37 °C for several hours. The seed culture was then transferred into minimal medium supplemented with casamino acids or different carbon sources (the same composition as the final experimental culture) and grew overnight as pre-cultures. The overnight pre-culture was inoculated into the same minimal medium at an initial OD_600_~0.01 to 0.02 as the final experimental culture. During the culturing process of the final experimental culture, 5–10 OD_600_ data points during exponential phase (generally within the OD_600_ range of 0.05~0.5) were measured by a Thermo Sci Genesys 50 spectrophotometer to calculate the exponential growth rate (in some cases, the OD measurement was done automatically by Biotek synergy H1 microplate reader). Note that for E. coli relA* OE strain (pLAS13-Ptac-relA*), the IPTG (purchased from GLPBIO) inducer was supplemented only at final culture stage (at OD_600_~0.02), a lower growth rate of *E. coli* would be generally observed after 2-3 generations of adaptation. For the glucose-acetate downshift experiments of *relA::kan, rho-15* mutant (Ts), seed culture and pre-cultures were incubated under 32 °C, the final culture was initially cultured at 32 °C in glucose minimal medium to OD_600_~0.2, then shifted to 42 °C for further ~1 h to partially disable the function of *rho* factor and then subjected to acetate downshift (also 42 °C).

### Nutrient downshift experiment

The nutrient downshift experiment was performed similarly as described in^11^. 15–20 mL bacteria cells were first exponentially growing in the 1st (preshift) medium to generally OD_600_~0.3 to 0.4, then culture was quickly transferred to the 2nd medium using vacuum filtration system and collected by a 0.22 µm filter membrane. The cells in the membrane were further washed twice by the prewarmed postshift M9 minimal medium (5–10 mL each time). The filter membrane was then quickly transformed into a sterilized petri dish and the cells were washed into the petri dish by 5 mL prewarmed postshift M9 minimal medium and further transferred into the flask with the postshift minimal medium (as time zero (*T*_0_)) at an initial postshift OD_600_~0.1 to 0.2 (the liquid culture in the petri dish was quickly measured for OD_600_ in order to estimate the volume of liquid culture that needed to be transferred) for further incubation and OD_600_ measurement (either by manual determination using spectrophotometer or automatic determination by microplate reader). The time cost of the whole nutrient downshift transfer procedure generally took less than 3 min. Note that the OD_600_ during postshift is calibrated as normalized OD_600_. For example, if the final OD just before downshift is 0.3, and the OD_600_ immediately determined after the medium transfer is 0.1. Then all the OD_600_ during postshift is multiplied by a factor of 3 as the normalized OD to obtain the full-range nutrient downshift growth curve (e.g., Fig. 1C).

### Carbon diauxie experiment

For the carbon diauxie experiments of *E. coli* and *V. natrigenes*, the overnight pre-cultures were grown in M9 glucose minimal medium. In the next day, the overnight pre-cultures were transferred into the final M9 minimal medium containing both glucose and the 2nd carbon source. Note that in the final culture medium, glucose, the 1st carbon, was generally supplemented at a low concentration, 0.05% for *E. coli* and 0.1% for *V. natriegens*, which were just 1/4 of their normal supplemented concentrations. The OD_600_ during the whole process of carbon diauxie experiment was automatically monitored by Biotek synergy H1 microplate reader.

### Lag-time quantification

For lag time quantification of the nutrient downshift using filtration method, the exponential range of the postshift medium (after the cells had completely resumed growth) was first analyzed by using exponential fitting (R square generally higher than 0.999) to get the exponential growth function (OD_600_ vs. time). The initial OD_600_ of postshift culture was designated as OD_ini_. Then with the exponential function and the OD_ini_, we could obtain the exact time point when exponential growth just resumed, *T*_resume_. The lag time, *T*_lag_ = *T*_resume_ – *T*_0_, where *T*_0_ is the time zero of postshift (the time when cells were just transferred into the postshift medium).

For lag time quantification in the case of carbon diauxie (exemplified in supplementary Fig. 8), we need to obtain the exponential functions of both preshift and postshift stage, corresponding to the exponential stage with 1st carbon and 2nd carbon, respectively. The average OD_600_ during the range of lag stage is taken as the OD_lag_. Then based on the OD_lag_ value and two exponential functions of both preshift and postshift stage, we could obtain two time points, *T*_preshift_ and *T*_postshift_, which denotes the end time point of the preshift exponential growth and the start time point of the postshift exponential growth. In this case, *T*_lag_ = *T*_postshift_ – T_preshift_.

### Measurement of cellular ppGpp pools

The measurement of ppGpp pools was similar as described in Ryals et al.^77^ with some modifications. 45 mL exponentially growing culture (OD_600_~0.3 to 0.4) was treated with 5 mL pre-chilled 1.8% formaldehyde and cooled on ice for 20–30 min. Cell pellets were harvested and further suspended in 0.5 mL 0.6 M KOH and lysed for 30 min on ice. KOH was then neutralized by addition of 15 μL of 85% phosphoric acid. The sample was further centrifuged at 10,000 × *g*, 3 min (4 °C) and the supernatant was taken. Before HPLC running, the sample should be filtered by a Corning HPLC membrane filter (No. 8161 or 8162). 20–50 μL samples were injected into an Agilent 1260 HPLC machine with a C18 column (250${\times }$4.6 mm, 5 μm) at a flow rate of 1 mL/min and monitored at 254 nm. The running procedures followed a gradient procedure: 0 to 28 min buffer A: B (82:18) increased linearly to buffer A: B (70:30). Buffer A: 0.03 M KH_2_PO_4_, 0.01 M tetrabutylammonium phosphate and adjusted to pH 3.8 by phosphoric acid. Buffer B: 100% acetonitrile. The ppGpp standard sample was bought from Tri-link.

### Measurement of transcription and translation kinetics during nutrient downshift

The protocol of transcription kinetics measurement was based on the recently established qRT-PCR approach^52,53,57^ and is described below again. Translation elongation rate measurement in Fig. 7 was obtained by the classical LacZ induction assay combined with Schelif plot square-root analysis with a 10-s calibration of the time cost of initiation steps (including IPTG penetration, LacI depression, transcription and translation initiation)^58,59^.

Two types of transcriptional kinetics were measured in this work: (1) the induction kinetics of related catabolite operons during specific carbon downshift (*lacZYA* for lactose downshift, *glpFK & glpD* for glycerol downshift and *aceBA* for acetate downshift), as shown in Fig. 6. (2) the induction kinetics of *lacZ* mRNA for measurement of the transcription elongation rate of *E. coli* during glucose to acetate downshift. For the first type of transcription kinetics, immediately after the transfer of preshift culture to the 2^nd^ carbon postshift medium (filtration method), 0.9 mL cell culture was withdrawn at a 1–2 min interval (depended on the exact growth condition) and transferred to 1 mL pre-chilled stop solution (60% ethanol, 2% phenol and 10 mM EDTA). Note that during the sample transfer process, carbon-free M9 minimal medium was used to wash the cells in the 0.22 µm filter to avoid earlier induction of specific catabolic operons. For the measurement of LacZ protein activity in the case of glucose to lactose downshift, 0.4 mL cell culture was also withdrawn at 1–3 min interval (depended on whether it is wild type or *relA*-deficient strain) and transferred into a precooled centrifuge tube (Nest Biotech) containing 10 μL 50 mg/mL chloramphenicol and thrown into liquid nitrogen immediately. For the second type of transcription kinetics, after transferring the cell culture from glucose to acetate, cell culture was first incubated for 5 min. 5 mM IPTG was then added to induce the expression of *lac* operon for measurement of both *lacZ* mRNA and LacZ proteins. Cell samples for both RNA and protein were taken similarly as described for the first type of experiments, however, at a 20 to 30-s interval. The protein samples could be directly stored at –80 °C for a few days before measurement of LacZ activity. Cell samples for RNA were pelleted at 4 °C at 10,000 × *g* for 2 min, flash frozen by liquid nitrogen and also stored at –80 °C for a few days before RNA extraction.

For RNA extraction, cell pellets were first lysed by 10 mg/mL lysozymes for 10 min at room temperature. Total cellular RNA of *E. coli* was then extracted using a bacterial RNA extraction kit (TianGen Biotech, Beijing). The cDNA synthesis (1 to 2 μg of total cellular RNA was applied) was performed with a first-strand cDNA synthesis reverse transcriptase kit (TianGen). Any contamination of cellular DNA was eliminated by Dnase I and gDnase (both supplied in the kit) during RNA extraction process or cDNA synthesis process, respectively. The qRT-PCR reaction was performed by an ABI QuantStudio 3 real-time Thermocycler using either PowerUp SYBR green Master mix (ABI) or Super-premix SYBR green Plus kit (Yeasen Biotech) according to the manual. The specificity of the primers was confirmed by the single-peak melting curve. The sequences of oligonucleotide primer are listed in Supplementary Table. The mRNA abundance of a sample taken immediately at time zero (referred to as ‘basal sample’), M (0), was set as “1”. The relative mRNA abundance at each time point, M(t), equals to 2^Ct(0) – Ct(*t*)^. For the measurement of transcription elongation rate of *lacZ* shown in Fig. 7, the linear range of the transcription kinetics data for different pairs of primers was fit with a linear line, being expressed as *y* = *a* × *x* – *b*, where “*a*” (the slope of the linear line) denotes the relative mRNA accumulation rate of each sub-regions. The transcription time of each mRNA sub-region, namely *T*_head_, *T*_mid_, *T*_tail_, equals to (1 + *b*)/*a* since “1” is the basal line value before mRNA induction. The transcription elongation rate of *lacZ* equals to 2808/(*T*_tail_ – *T*_head_), where 2808 denotes the distance (nt) between the tail region (detected by P3105) and head region of *lacZ* (detected by P297). In the case of *relA*-deficient strain, the tail region of *lacZ* mRNA was not induced due to strong premature transcription termination (PTT). The transcription elongation rate could be analyzed by the data of the middle region (P1578) and head region (P297 primer), equaling to 1281/(*T*_mid _– *T*_head_). Note that, for the case of wild type strain, data obtained by 2808/(*T*_tail_ – *T*_head_) is comparable to that obtained by 1281/(*T*_mid_ – *T*_head_).

### Proteomics by 4D-label-free mass spectrometry

For proteomic studies, ~30 mL exponential culture of *E. coli* (OD600~0.3) was transferred into a precooled 50 mL centrifuge tube (Nest Biotech), and collected by centrifuge (4 °C). The cell pellets were washed twice by PBS and stored at –80 °C freezer prior to proteomic analysis. The proteomics was based on 4D label-free mass spectrometry approach^78^, which was performed by Jingjie PTM Biolabs (Zhou Hang). The experimental procedure of 4D label-free method was described as below: the cell pellets were subject to ultra-sonication in lysis buffer (8 M Urea 8 M urea, 1% Triton X-100, 10 mM DTT, 1% protease inhibitor cocktail and 2 mM EDTA). The cell debris was then removed by centrifuge (4 °C, 12,000 × *g*) for 10 min. The supernatant was transferred into a new centrifuge tube for protein concentration measurement using BCA kit. Protein samples were next subject to trypsin digestion. For digestion, protein samples were first pelleted by 20% TCA at 4 °C for 2 h and then collected with centrifuge (4500 × *g*) for 5 min. The precipitates were washed for twice using precooled acetone and further added with 200 mM TEAB. Trypsin was then added at 1:50 trypsin-to-protein mass ratio for digestion overnight. The solution was reduced with 5 mM DTT for 30 min at 56 °C and alkylated with 11 mM iodoacetamide for 15 min at room temperature in darkness. Finally, the peptides were desalted by C18 SPE column. Solvent A (0.1% formic acid, 2% acetonitrile) and Solvent B (0.1% formic acid, 100% acetonitrile) were used for following peptides separation and UPLC procedures. The peptides were dissolved in solvent A and separated by the NanoElute UPLC system. The flow setting of UPLC is as follows: 0–43 min, 6%–22%B; 43–55 min, 22%–30%B; 55–58 min, 30%–80%B; 58–61 min, 80%B; flow rate: 450 nL/min. After the separation by UPLC, the peptide was set into Capillary ionization source for ionization and further analyzed by timsTOF Pro mass spectrometry system. The electrospray voltage applied was set at 1.6 to 1.8 kV. Both the original peptide ion and its secondary fragments were detected and analyzed by high-resolution TOF. The *m*/*z* scan range was 100 to 1700 for full scan. Precursors with charge states 0 to 5 were selected for fragmentation, and 10 PASEF-MS/MS scans were acquired per cycle. The dynamic exclusion was set to 30 s. The mass spectra data were searched against the SwissProt *E. coli* K-12 databases and analyzed by Maxquant v1.6.15.0 software^39^, which gave the information of both LFQ intensity and iBAQ intensity. The information of the relative abundance of each protein across different conditions was given by LFQ intensity. The mass proteome fraction (absolute abundance) of individual proteins was obtained using the iBAQ intensity of each protein to multiply the molecular weight (MW) (we referred to as “iBAQ mass” in Supplementary Data) and further normalized by the sum of the whole proteome (as iBAQ is a proxy of the copy number of each protein). The iBAQ mass of individual proteins, together with the gene locus-tag were submitted to proteomaps website to obtain the KEGG resource allocation map of *E. coli* cells^40^.

### Statistics and reproducibility

No statistical method was used to predetermine sample size and no data were excluded from the analyses. The experiments were not randomized. The Investigators were not blinded to allocation during experiments and outcome assessment. As many as replicates as possible were done for the papers. Most key data such as lag times, growth rates have generally been repeated for at least three times. See the figure legends for details. The deviations between biological replicates are small (generally within 10-15%) and are thus highly reproducible and sufficient to draw solid conclusions. The data analysis and presentation are done with Graphpad Prism 8.0.

### Reporting summary

Further information on research design is available in the Nature Portfolio Reporting Summary linked to this article.

## Supplementary information

Supplementary Information

Description of Additional Supplementary Files

Supplementary Data 1-9

Reporting Summary

## Data Availability

The mass spectrometry proteomics data have been deposited to the ProteomeXchange Consortium via the PRIDE partner repository with the dataset identifier PXD035675. The core data of proteomics in the paper are also provided in the supplementary data file. Source data are provided with this paper.

## Source data

Source Data
