# Supplementary material for: Stringent response ensures the timely adaptation of bacterial growth to nutrient downshift
Source: Nat Commun. 2023 Jan 28;14:467. doi: 10.1038/s41467-023-36254-0 (PMC9884231; doi:10.1038/s41467-023-36254-0)
Supplement: Supplementary file 1 — Supplementary Information [file 41467_2023_36254_MOESM1_ESM.pdf]

Supplementary Information：Stringent response ensures the timely adaptation of bacterial growth to nutrient downshift

Manlu Zhu[1]*, Xiongfeng Dai[1]*

[1]Hubei Key Laboratory of Genetic Regulation and Integrative Biology, School of Life Sciences, Central China Normal University, Wuhan, Hubei province, China

*correspondence: zhumanlu@ccnu.edu.cn; daixiongfeng@ccnu.edu.cn

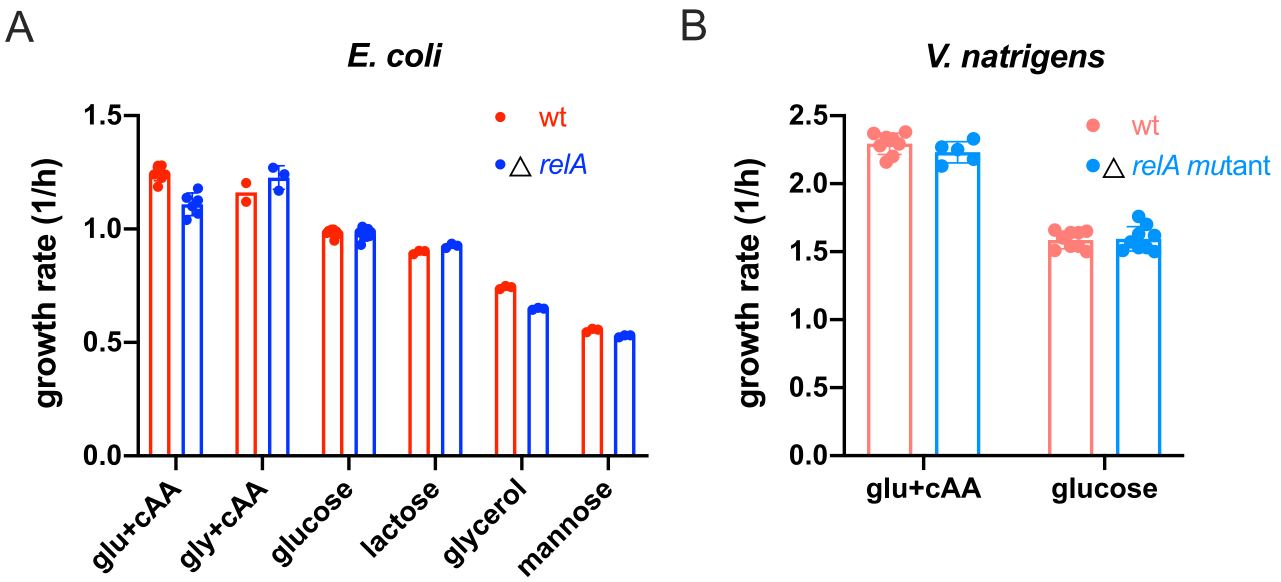

Supplementary Fig. 1 The exponential growth rate of *E. coli* (WT vs *relA* deficient strain) and *V. natriegens* (WT vs *relA* deficient strain) under various kinds of nutrient sources. Error bars are the standard deviations of several biological replicates. n=3 for lactose, glycerol and mannose conditions of *E. coli*. n=9 for glucose condition of *E. coli*. n=8 and 6 for glu+cAA condition of *E. coli* wild type strain and *relA*-deficient strain, respectively. n=2 and 3 for gly+cAA condition of *E. coli* wild type strain and *relA*-deficient strain, respectively. n=8 and 5 for glu+cAA condition of *V. natriegens* wild type strain and *relA*-deficient strain, respectively. n=9 for glucose condition of *V. natriegens*. Source data are provided as a Source Data file.

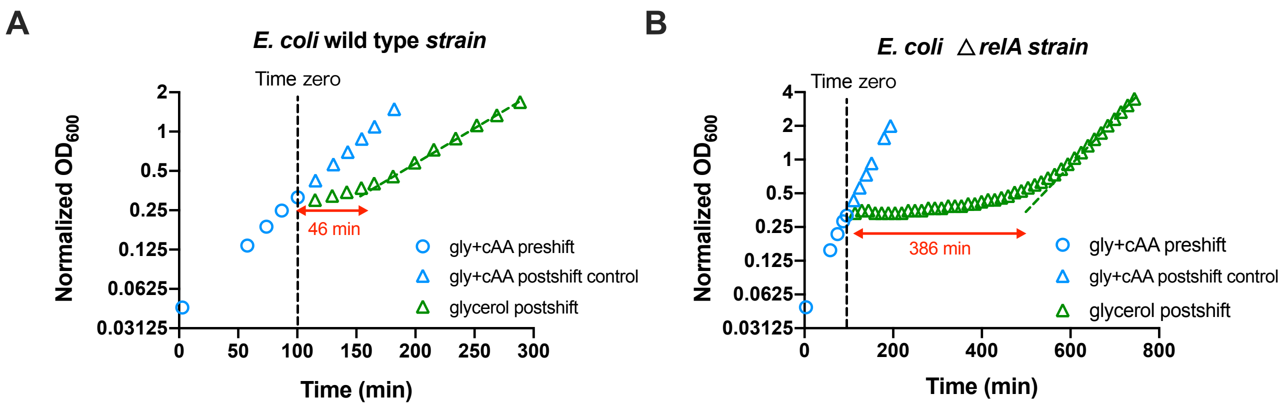

Supplementary Fig. 2 Growth curve and lag time of *E. coli* during transition from glycerol plus casamino acid (gly+cAA) medium to amino acid-free glycerol (gly) minimal medium. (A) Growth curve and lag time of wild type *E. coli* cells during transition from glycerol cAA medium to glycerol minimal medium. (B) Growth curve and lag time of *E. coli relA* deficient mutant during transition from glycerol cAA medium to glycerol minimal medium. Source data are provided as a Source Data file.

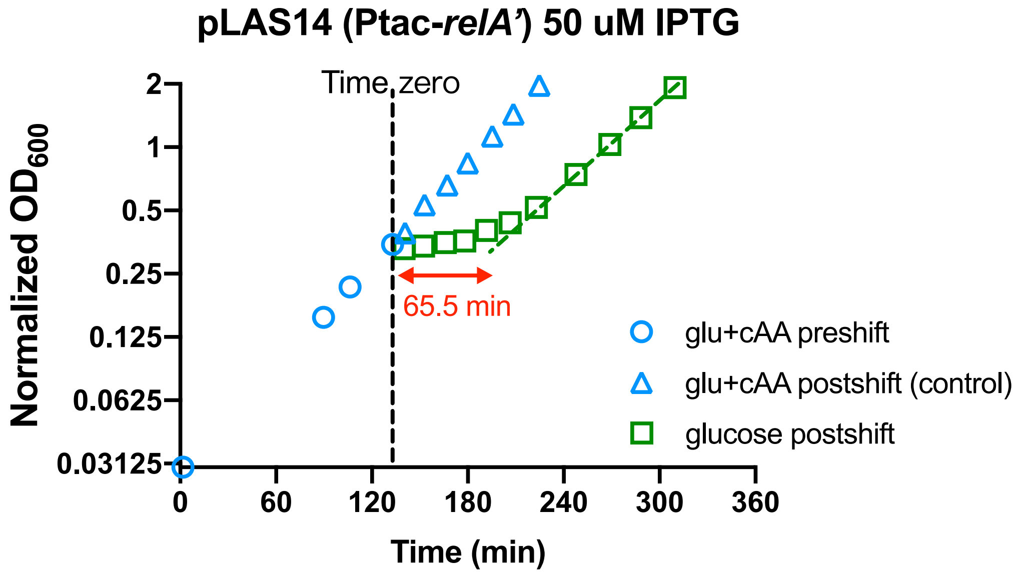

Supplementary Fig. 3 Growth and lag time of *E. coli* (pLAS14) during transition from glucose cAA medium to glucose minimal medium. *E. coli* was transformed with the pLAS14 vector, in which an inactive *relA*' (encoding the N-terminal 1-331 aa of the native *relA*) was driven by the IPTG-inducible *Ptac* promoter. IPTG was added at a concentration of 50 µM. Source data are provided as a Source Data file.

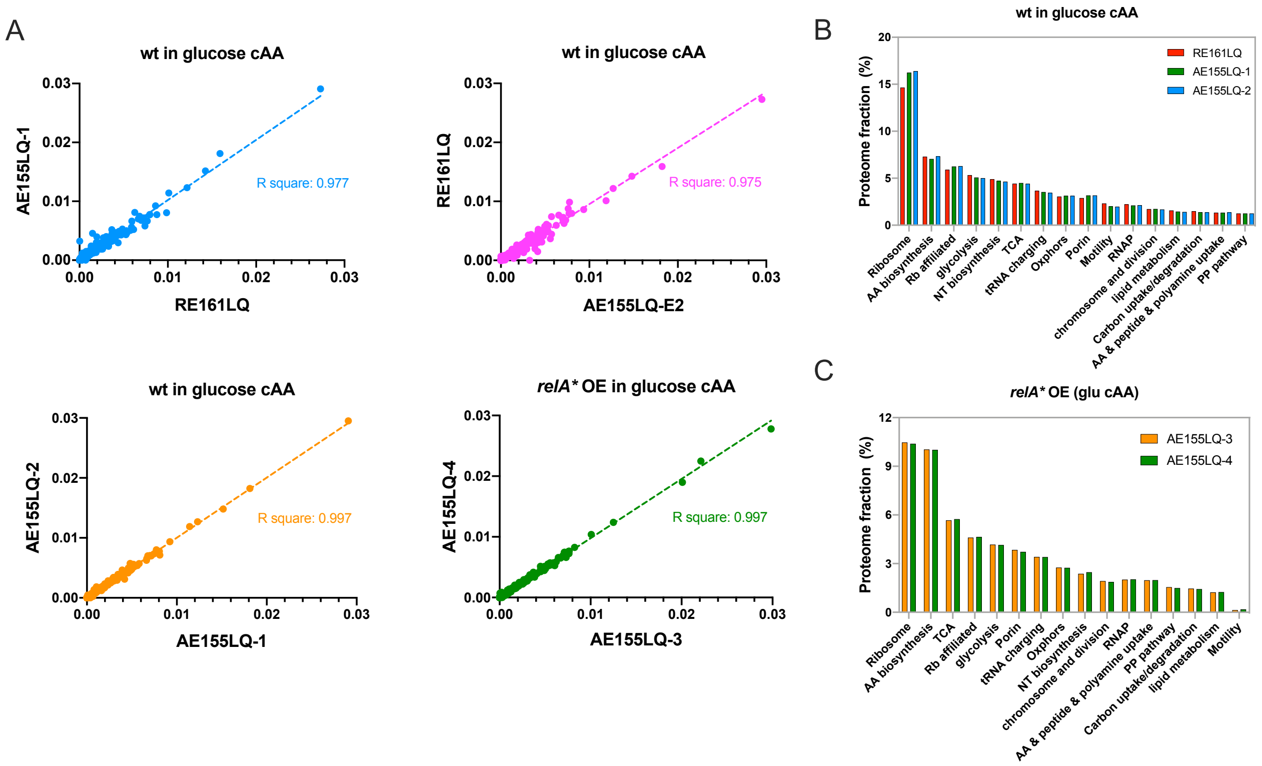

Supplementary Fig. 4 Reproducibility of the proteomics data measured by 4D label-free mass spectrometry. The condition of wild type *E. coli* cells growing in glucose cAA medium had been done for three times (samples of three biological replicates), see supplementary data for details. Two samples of them were analyzed together in the AE155LQ project (E1 and E2), and the other one was done in the RE161LQ project (WT_0 sample). The condition of *relA** OE (*E. coli* pLAS13-*relA**strain in glucose cAA medium supplemented with 30 μM IPTG) had been done twice (samples of two biological replicates) and were analyzed together in the AE155LQ project (E3 and E4). We analyzed the mass proteome fraction of each individual proteins using the information of iBAQ intensity×MW (molecular weight) normalized by the sum of the whole proteome (see method and supplementary data). We then plotted different replicates against with each other and confirmed the high reproducibility of different replicates (four subpanels of panel A). In addition, when we compare the proteome fraction of a whole functional sectors, all the replicates show very similar results with each other (panel B and panel C) with the standard deviation being within 10%. Source data of iBAQ mass of panel A is provided in supplementary data 2 and 5. Source data are provided as a Source Data file.

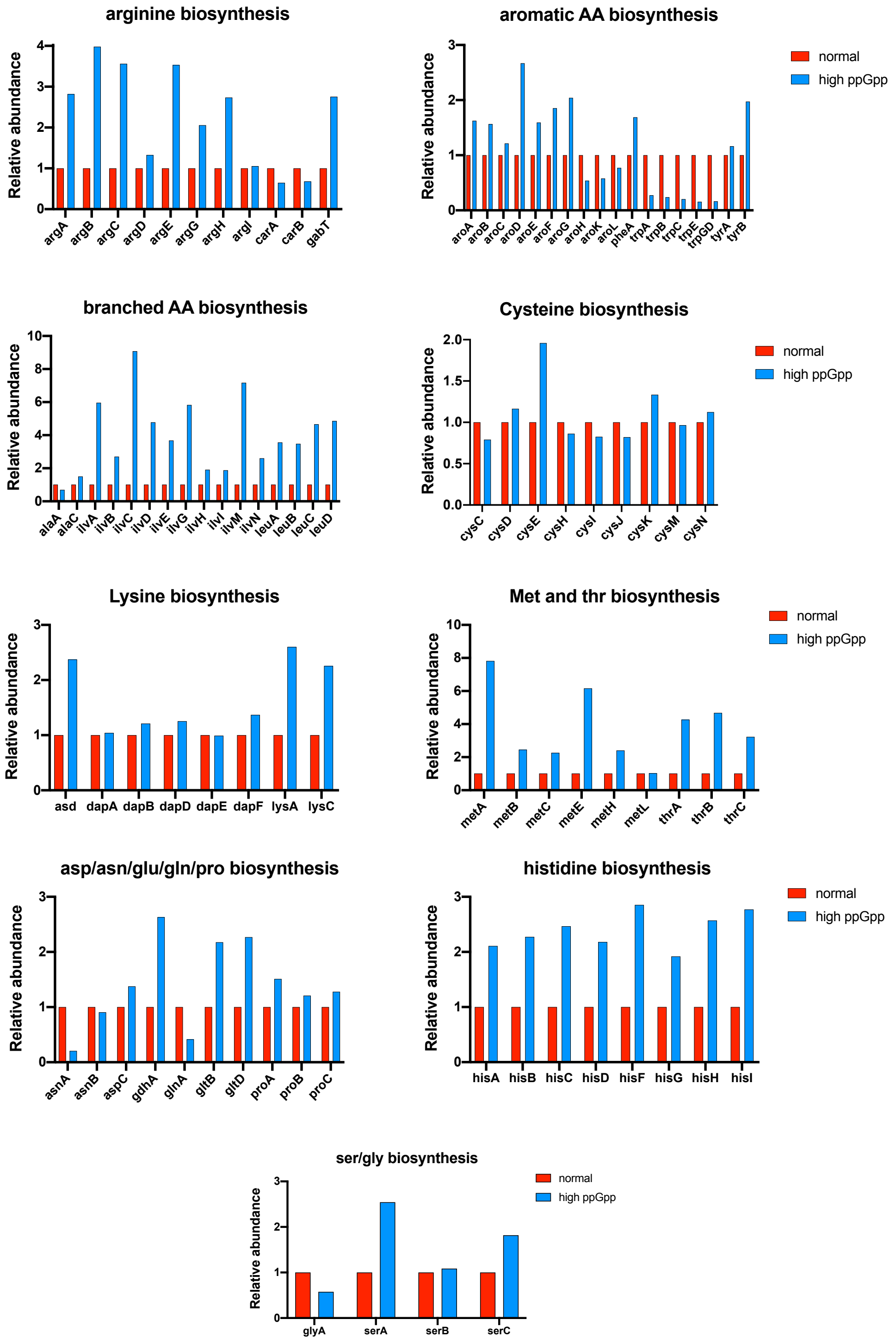

Supplementary Fig. 5 Relative abundances of individual amino acid biosynthesis proteins. The proteome abundances of *E. coli* wild type strain and RelA* OE strain (high ppGpp) during exponential growth in glucose cAA medium were compared by 4D label-free mass spectrometry. The data of relative abundance is given by the LFQ intensity. The medium of *E. coli* (pLAS13-*relA**) strain was supplemented with 30 µM IPTG. This condition is thus referred to as "high ppGpp". Source data are provided as a Source Data file.

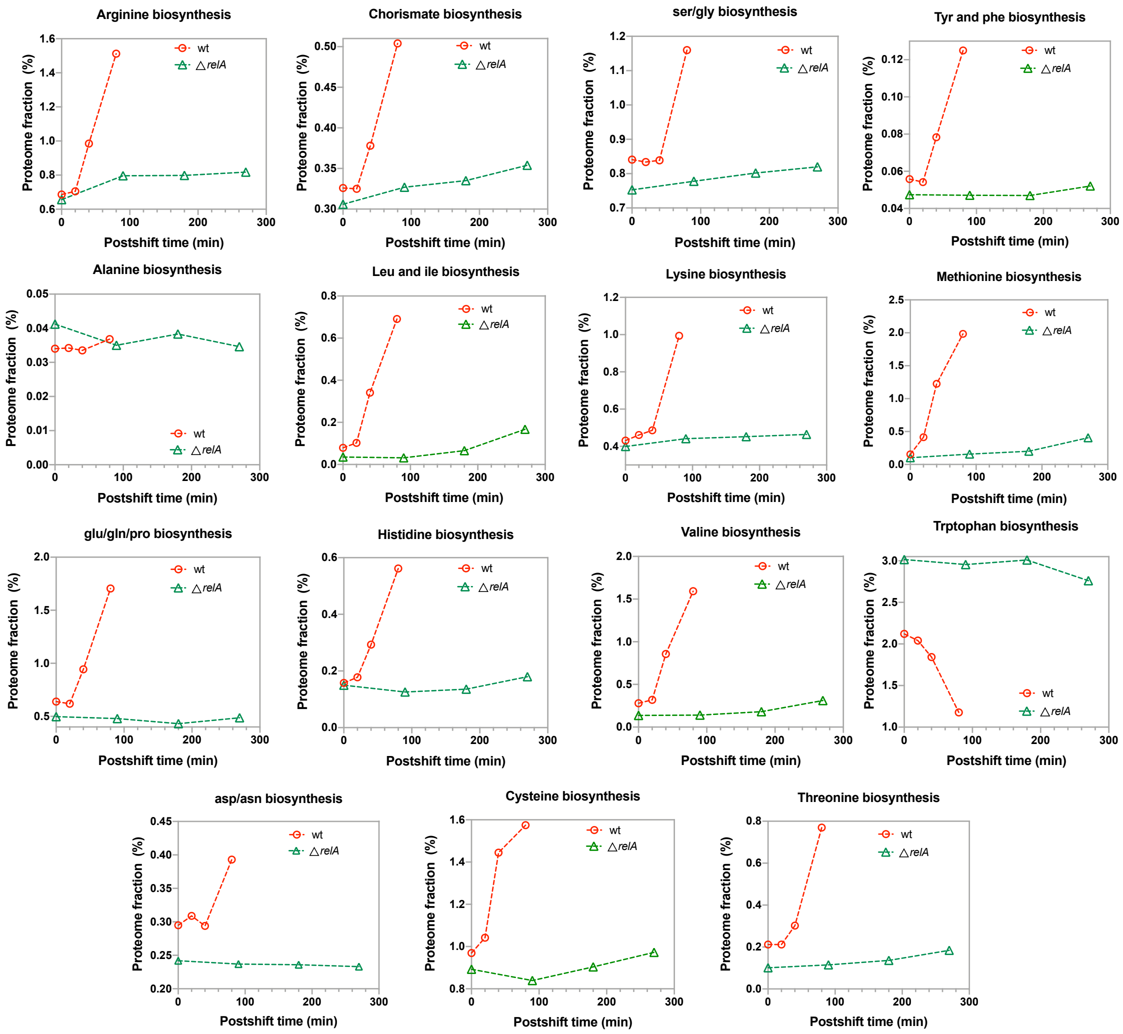

Supplementary Fig. 6 Dynamic changes in the proteome fractions of each AA biosynthetic subgroup. Most subgroups of amino acid biosynthesis in wild type strain generally follows the same increasing trend as the whole amino acid biosynthesis sector except alanine biosynthesis and tryptophan biosynthesis. The abundance of alanine biosynthesis sector keeps constant during AA downshift. Unlike other amino acid subgroups, tryptophan biosynthetic pathway in wild type strain exhibits a clear decreasing trend during AA downshift, as also found in the case of RelA* overexpression (Figure 3G). Instead, its abundance is largely maintained at a constant level in the *relA*-deficient strain. Similarly, in the case of RelA* OE, the level of tryptophan biosynthesis sector drops by 75% (Figure 3G) instead of increasing like other amino acid subgroups. This effect might be complicated by the fact that tryptophan is absent in the component of casamino acid. In such case, tryptophan biosynthesis has already been maximally induced. Indeed, the proteome fraction of tryptophan biosynthesis reaches 2%, accounting for nearly 30% of the total AA biosynthesis sector. Therefore, the trend of tryptophan biosynthesis here could be altered if tryptophan is supplied into the medium. Source data are provided as a Source Data file.

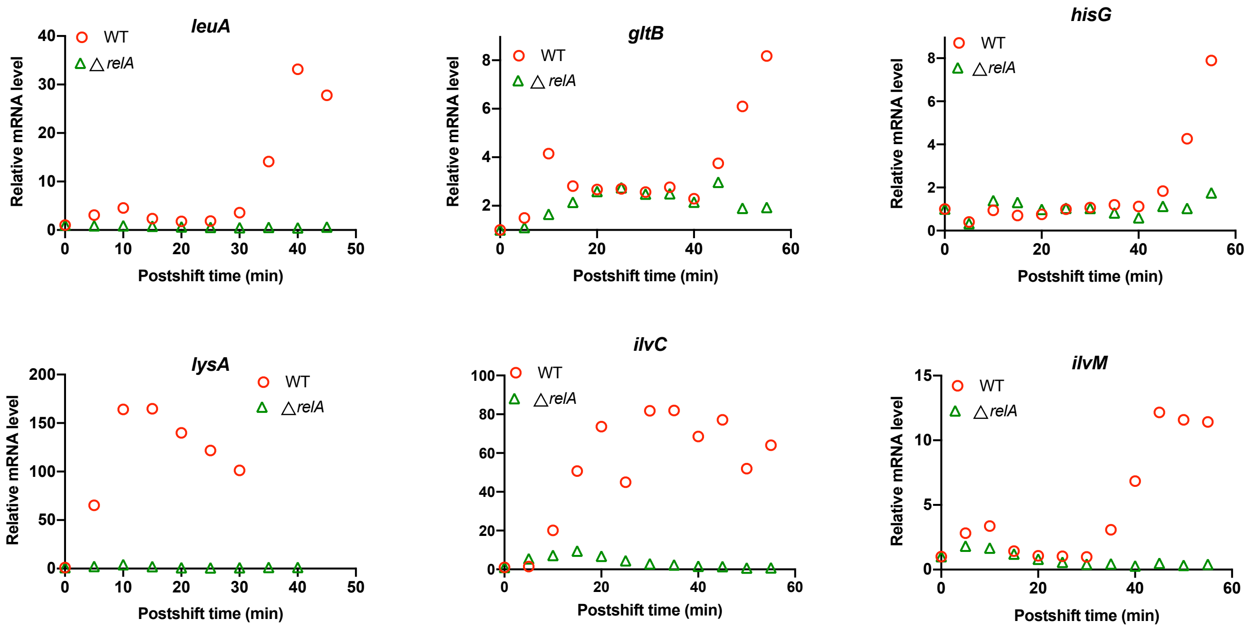

Supplementary Fig. 7 Dynamic change in the mRNA abundances of several amino acid biosynthetic genes during amino acid (AA) downshift. Conditions are exactly the same as shown in Figure 4. The six genes are all located at the 5' proximal or upper region of their own operons (except that *ilvM* is the second gene of *ilvGMEDA* operon). The qPCR primers (attached below) detect the 5' regions of these operons, and thus could denote the status of transcription initiation of these genes. qPCR primer sequences are listed in supplementary table. Source data are provided as a Source Data file.

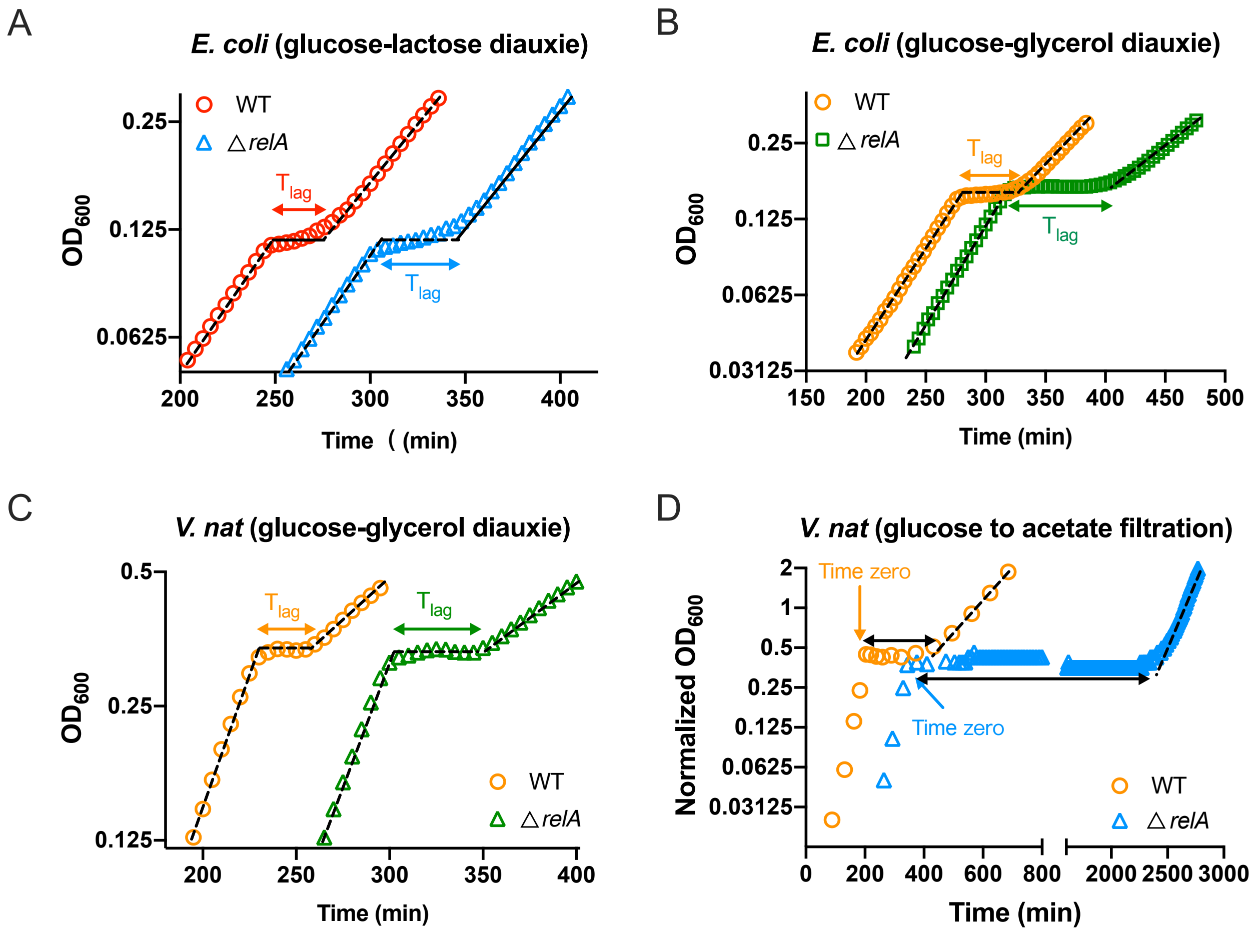

Supplementary Fig. 8 Growth data of carbon downshift experiments of *E. coli* and *V. natrigens*. (A) glucose-lactose diauxie of *E. coli.* (B) glucose-glycerol diauxie of *E. coli* (C) glucose-glycerol diauxie of *V. natriegens*. (D) glucose to acetate downshift of *V. natriegens* (by filtration method). Source data are provided as a Source Data file.

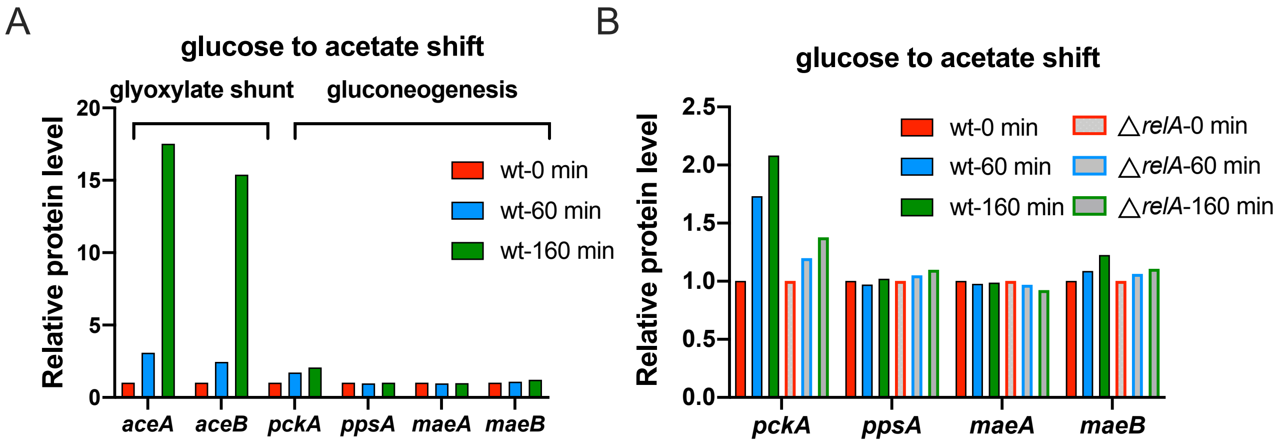

Supplementary Fig. 9 The relative abundances of glyoxylate shunt and gluconeogenesis proteins of *E. coli* at different time points after shifting from glucose to acetate medium (at 0 min, 60 min and 160 min, respectively). (A) wild type cells. (B) wild type vs *relA*-deficient strain for the four gluconeogenesis proteins. Source data are provided as a Source Data file.

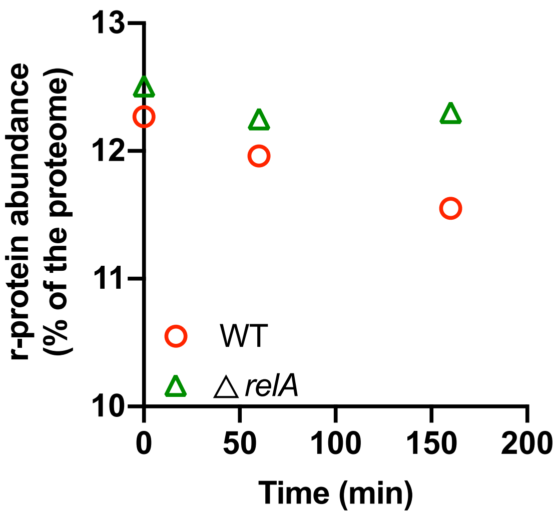

Supplementary Fig. 10 The dynamic change in the proteome fraction of ribosomal proteins (r-protein) during glucose to acetate downshift at 0 min, 60 min and 160 min of postshift for both wild type strain (lag time: 193 min) and *relA*-deficient strain (lag time: 517 min). Source data are provided as a Source Data file as well as supplementary data 9.

Supplementary Table: oligonucleotide primers used in this study

primer name	sequence (5' to 3')
lac operon	
P297-F	CAGCCTGAATGGCGAATGG
P297-R	CGACGACAGTATCGGCCTC
P898-F	CGTGACTACCTACGGGTAACAG
P898-R	GCATAACCACCACGCTCATC
P1578-F	GCTGGATCAAATCTGTCGATCC
P1578-R	GGAAGGGCTGGTCTTCATCC
P3105-F	GGCACATGGCTGAATATCGACG
P3105-R	GACACCAGACCAACTGGTAATGG
lacY-q-F2	TTATCTGGTGCTGGGTCTGGT
lacY-q-R2	CGACTTCATTCACCTGACGACG
glpFK operon	
glpF-head-F	AGTGGGAAATCAGTGTCATTTGGG
glpF-head-R	TGGTAACAGCGGGATTAAGATGC
glpF-tail-F	GACATTCCTTACTTCCTGGTGCC
glpF-tai-R	CAGTTTGCGGTAGGCAAATGC
glpK-head-F	TATATCGTTGCGCTCGACCAG
glpK-head-R	GACACGCTAATGATATTGGCATCG
glpK-tail-F	TTGGCTTCTGGCAGAACCTC
glpK-tail-R	CTCAGTGGTTTCGATGCCTGG
glpD	
glpD-head-F	TTATCCGTGCTGATGCTGGAG
glpD-head-R	GAATTCATAGTGCTCAAGGTAGC
glpD-mid-F	TGGCATTCGCCTGATCAAAGG
glpD-mid-R	CACGGGATCACGAACACAATACG
glpD-tail-F	AACAAGGCATGTGGCTAAATGC
glpD-tail-R	GCGATAACCTCTGCTGCGTATAC
aceBA	
aceB-head-F:	CAACAACAACCGATGAACTGGC
aceB-head-R:	CAGAAGTTTATTGCGTTGTGGCG
aceB-tail-F:	AACGTTGAGCAATGGCAAACC
aceB-tail-R:	GGAGAAACGTTCTTCGCCCAG
aceA-head-F:	ACAGAAAGAGTGGACTCAACCG
aceA-head-R:	CAGCGTGCATTCAGGATTGAC
aceA-tail-F:	AGGGTATGAAGCACTACGTTGAG
aceA-tail-R:	CGAAGTAACCTGTACCCACTTCC
amino acids biosynthesis	
leuA-F	CACCAGCCAGTTAGTTAGCCA
leuA-R	GGTGTATACCGGAGGAGTGTG
gltB-F	CGCATCGTTGAAGAAGAACTGC

gltB-R	CTTCGTTAGTGGGGACATCACG
hisG-F	CATGCCGATTGATATTCTGCGC
hisG-R	AGCACGTTTTCGCCGATAATC
lysA-F	ATTCACTGTTCAGCACCGATACC
lysA-R	CGAATAATTTGCGCATCGTAGACC
ilvC-head-F	AATACACTGAATCTGCGCCAGC
ilvC-head-R	TTTACCCTGAAGGTAGCTCGC
ilvM-head-F	TGCAACATCAGGTCAATGTATCGG
ilvM-head-R:	TTCATTGAGCAGACGTGGAAACC
