# Supplementary material for: Stringent response ensures the timely adaptation of bacterial growth to nutrient downshift
Source: Nat Commun. 2023 Jan 28;14:467. doi: 10.1038/s41467-023-36254-0 (PMC9884231; doi:10.1038/s41467-023-36254-0)
Supplement: Supplementary file 2 — Description of Additional Supplementary Files [file 41467_2023_36254_MOESM2_ESM.pdf]

Description of Additional Supplementary Files

File Name: Supplementary Data 1
Description: AE155LQ project (wild type vs ppGpp overproduction during exponential growth). A systematic annoation of proteomic data of AE155LQ provided by the Jingjie PTM Biolabs. The relative abundances of each protein (column AD to AG) corresponds to exactly the data of LFQ intensity (column Z to AC). It is obtained by using the LFQ intensity of each condition to be normalized by the average of the LFQ intensity of all conditions of each protein.

File Name: Supplementary Data 2
Description: Absolute abundance of each individual protein wtih the information of iBaq intensity. Maxquant raw data gives the information of iBAQ intensity, which is a proxy of the copy number of each protein. We then use the iBAQ intensity of each protein to multiply its molecular weight (MW) to obtain the iBAQ mass. The proteome fraction of each protein (or a protein function sector) is obainted by normalizing its value by the sum of the whole proteome.

File Name: Supplementary Data 3
Description: The proteome fraction of each proteome function sector using the information of iBAQ mass in Supplementary Data 2.

File Name: Supplementary Data 4
Description: RE161LQ (WT vs relA deficient strain during AA downshift). A systematic annoation of proteomic data of RE161LQ provided by the Jingjie PTM Biolabs. The relative abundances of each protein (column AX to BE) corresponds to exactly the data of LFQ intensity (column AP to AW). It is obtained by using the LFQ intensity of each condition to be normalized by the average of the LFQ intensity of all conditions of each protein.

File Name: Supplementary Data 5
Description: The iBAQ intensity and iBAQ mass of the RE161LQ project, being similar to Supplementary Data 2.

File Name: Supplementary Data 6
Description: The proteome fraction of amino acid biosynthesis sector (the whole sector as well as individual subgroup) and ribosomes for wild type strain and *relA* deficient strain during AA downshift.

File Name: Supplementary Data 7
Description: The proteome fraction of each proteome function sector for the RE161LQ (WT_0 sample) using the information of iBAQ mass in Supplementary Data 5. WT_0 sample in RE161LQ is a 3rd biological replicate sample of AE155LQ E1 and E2 sample.

File Name: Supplementary Data 8

Description: A systematic annoation of proteomic data of RE235LQ provided by the Jingjie PTM Biolabs. The relative abundances of each protein (column AN to AS) corresponds to exactly the data of LFQ intensity (column AH to AM). It is obtained by using the LFQ intensity of each condition to be normalized by the average of the LFQ intensity of all conditions of each protein.

File Name: Supplementary Data 9

Description: The proteome fraction of ribosomes for wild type strain and *relA* deficient strain during glucose to acetate downshift. The iBAQ intensity and iBAQ mass of the RE235LQ are shown.
